# Global Prevalence and Specificity of Red Blood Cell Antibodies in Blood Donors: A Systematic Review and Meta-Analysis

**DOI:** 10.3390/diseases14080298

**Published:** 2026-08-18

**Authors:** Thitinat Duangchan, Moltira Promkan, Susan D. Kraner, Nurdina Charong

**Affiliations:** 1 Department of Medical Technology, School of Allied Health Sciences, Walailak University, Nakhon Si Thammarat 80160, Thailand; thitinat.du@wu.ac.th; 2 Hematology and Transfusion Science Research Center, Walailak University, Nakhon Si Thammarat 80160, Thailand; 3 Department of Clinical Microscopy, Faculty of Medical Technology, Mahidol University, Nakhon Pathom 73170, Thailand; 4 Sanders-Brown Center on Aging, University of Kentucky College of Medicine, Lexington, KY 40536, USA

**Keywords:** alloantibody, red blood cell antibodies, blood donors, transfusion medicine, systematic review, meta-analysis

## Abstract

**Background:** Red blood cell (RBC) antibody screening in blood donors is a key component of transfusion safety. However, reported prevalence and antibody specificities vary widely across studies, and a consolidated global estimate remains limited. This study aimed to quantify the prevalence of RBC antibody detection among blood donors, identify factors associated with variation in reported prevalence, and summarize antibody specificities. **Methods:** This systematic review and meta-analysis was conducted in accordance with PRISMA 2020 guidelines. PubMed, MEDLINE, Embase, Scopus, and Google Scholar were searched from inception to 27 August 2025 for observational studies reporting RBC antibody detection in blood donors. Methodological quality was assessed using the JBI Critical Appraisal Checklist for Studies Reporting Prevalence Data. Pooled prevalence estimates were synthesized using random-effects models. Subgroup analyses, meta-regression, leave-one-out sensitivity analyses, and publication-bias assessments were performed. Associations between ABO blood group and RBC antibody detection were summarized using pooled odds ratios. The review protocol was registered in PROSPERO (CRD420251181987). **Results:** Forty-one studies comprising 43 reports and 3,561,088 blood donors from 18 countries were included. The pooled prevalence of RBC antibody detection was 0.2% (95% CI: 0.2–0.3%), with substantial between-study heterogeneity (*I*^2^: 99.61%) and a 95% prediction interval of 0.0–1.4%. Prevalence was higher in female donors than in male donors (0.5% vs. 0.1%). Publication year was inversely associated with reported prevalence, although temporal and methodological effects could not be separated. An exploratory analysis based on six studies showed higher estimated odds of antibody detection among blood group AB donors (OR: 1.20, 95% CI: 1.07–1.34). Among clinically significant antibodies, Rh and Kell specificities were predominated. Anti-Mi^a^ was prominent in Southeast Asian populations. **Conclusions:** RBC antibody detection among blood donors was uncommon overall but varied considerably across settings. Interpretation is limited by substantial methodological heterogeneity, uneven geographic representation, and incomplete reporting of donor characteristics. The pooled estimate should be interpreted as an international benchmark rather than a uniform prevalence applicable to individual blood services. This large-scale synthesis may inform context-specific donor antibody screening, confirmatory testing, component management, and hemovigilance policies.

## 1. Introduction

Blood donation is a vital component of healthcare systems worldwide, supporting the management of trauma, surgery, malignancy, and chronic diseases [1]. Although blood transfusion is a life-saving treatment, its clinical effectiveness depends on both infectious and immunological safety. The presence of unexpected RBC antibodies in donor blood may have clinical consequences when antibody-containing components are transfused to recipients expressing the corresponding RBC antigens [2]. Unexpected RBC antibodies include antibodies directed against antigens outside the ABO system and may be naturally occurring or develop following immune stimulation, particularly after pregnancy or transfusion [3,4]. Their immunologic characteristics vary according to antibody specificity. Antibodies within the Rh, Kell, Kidd, and Duffy blood group systems are predominantly immune-stimulated and commonly of the IgG class, whereas antibodies in Lewis and P1PK systems are usually naturally occurring and often of the IgM class [5]. Antibodies within the MNS system are more heterogeneous. Anti-M and anti-N are often naturally occurring, whereas anti-S and anti-s are predominantly immune-stimulated [6,7]. Other antibodies, including anti-Mi^a^, may occur naturally or following immune stimulation [8]. Importantly, clinical significance is not determined solely by immunoglobulin class or presumed origin, but also depends on antibody specificity, thermal amplitude, titer, complement activity, and reactivity at 37 °C or in the antiglobulin phase [6].

RBC antibody screening is performed in routine blood transfusion services as a critical step to ensure transfusion safety. Detection of unexpected antibodies can guide appropriate component management and reduce the potential risk associated with passive transfer of clinically important antibodies to recipients [9]. Reported prevalence varies widely among blood donor populations and geographical regions, reflecting differences in RBC antigen frequencies, donor characteristics, laboratory methods, and screening practices. For example, a study of 47,450 healthy blood donors in India reported an alloantibody prevalence of 0.09% [10], whereas another large Indian donor study involving 166,803 donors reported RBC antibodies in 0.17% of donors [11]. Sex-related differences have also been reported, with several donor studies observing a higher prevalence among female donors, potentially reflecting differences in sensitizing exposures such as pregnancy [12,13]. Studies from some Asian populations have reported relatively frequent anti-M, anti-Le^a^, anti-Mi^a^, or anti-E [14,15], whereas Rh and Kell antibodies are prominent in many other populations [16,17]. These variations highlight the difficulty of applying prevalence estimates from individual populations to other blood services and underscore the need for a systematic synthesis of global donor data.

This systematic review and meta-analysis aimed to estimate the pooled prevalence of RBC antibody detection among blood donors, summarize reported antibody specificity patterns, and explore variation according to sex, geographic region, publication year, laboratory method, and ABO blood group. This review provides an international benchmark for RBC antibody detection and may inform context-specific donor screening, confirmatory testing, component-management, and transfusion-safety policies.

## 2. Materials and Methods

### 2.1. Protocol Registration

This systematic review and meta-analysis was conducted in accordance with the Preferred Reporting Items for Systematic Reviews and Meta-Analyses (PRISMA) 2020 guidelines. The completed PRISMA 2020 checklist is provided in Supplementary Table S1. The protocol was registered prospectively in the International Prospective Register of Systematic Reviews (PROSPERO) under the registration number CRD420251181987.

### 2.2. Search Strategy

A comprehensive and systematic literature search was conducted in PubMed, MEDLINE, Embase, Scopus, and Google Scholar from database inception to 27 August 2025, with no language restrictions applied. The search strategy combined controlled vocabulary and free-text terms related to blood donors and unexpected red blood cell antibodies. The complete search strategies are provided in Supplementary Table S2.

### 2.3. Study Selection

Observational studies reporting the prevalence of RBC alloantibodies among blood donors were eligible for inclusion. Studies were excluded if they included only patient populations or transfusion recipients, or if they were case reports, reviews, conference abstracts, animal studies, or did not report both the number of alloantibody-positive donors and the total number of donors screened. All retrieved records were imported into Rayyan reference management software, and duplicate records were removed prior to screening. Two reviewers (T.D. and N.C.) independently screened titles and abstracts for eligibility, followed by full-text assessment of potentially relevant articles. Any discrepancies were resolved through discussion and consensus.

### 2.4. Data Extraction

Data extraction was performed independently by two reviewers (T.D. and N.C.) using a standardized data extraction form developed in Microsoft Excel (Microsoft Corporation, Redmond, WA, USA). Extracted data were categorized into study characteristics and study outcomes. The study characteristics included first author, year of publication, country, sample size, mean age, sex distribution, and antibody detection methods. The study outcome variables included number of donors screened for antibodies, number of donors positive for alloantibodies and autoantibodies, distribution of antibody specificities, and frequencies of multiple alloantibodies. Any disagreements during the data extraction process were resolved through discussion and consensus between the reviewers.

### 2.5. Quality Assessment of Included Studies

The methodological quality of included studies was assessed independently by two reviewers (T.D. and N.C.) using the Joanna Briggs Institute (JBI) Critical Appraisal Checklist for Studies Reporting Prevalence Data [18], which evaluates nine methodological domains. Each domain was rated as yes, no, unclear, or not applicable. Any disagreements between reviewers were resolved through discussion and consensus. For descriptive interpretation, overall study quality was categorized as low, moderate, or high based on the number of domains rated as yes (low quality: 0–3, moderate quality: 4–6, high quality: 7–9) [19]. These categories were used to support interpretation and were not used as exclusion criteria.

### 2.6. Statistical Analysis

The overall prevalence of RBC antibody detection was estimated using a DerSimonian–Laird random-effects meta-analysis of logit-transformed proportions. Pooled estimates were back-transformed to the proportion scale and reported with 95% confidence intervals (CIs). A 95% prediction interval was also calculated. Between-study heterogeneity was assessed using the *I*^2^ statistic. Meta-regression analysis was performed using year of publication as a study-level covariate. Subgroup analyses were conducted according to antibody detection method, geographic region, and publication period. Sex-specific prevalence estimates were calculated separately for male and female donors. Association between ABO blood group and RBC antibody detection was evaluated using pooled odds ratios (ORs) with 95% CIs under fixed- or random-effects models. Leave-one-out sensitivity analyses were performed to examine the influence of individual studies on the overall pooled estimates. Small-study effects were assessed using funnel-plot inspection and Egger’s regression test. Pooled proportions of individual RBC antibody specificities were also estimated among antibody-positive donors. All statistical analyses were performed using Stata software (version 18.5; StataCorp LLC, College Station, TX, USA). Effect estimates were reported with 95% CIs and exact *p* values where applicable. Results were interpreted according to the magnitude, direction, and precision of the estimates [20].

## 3. Results

### 3.1. Study Selection

**Figure 1:**
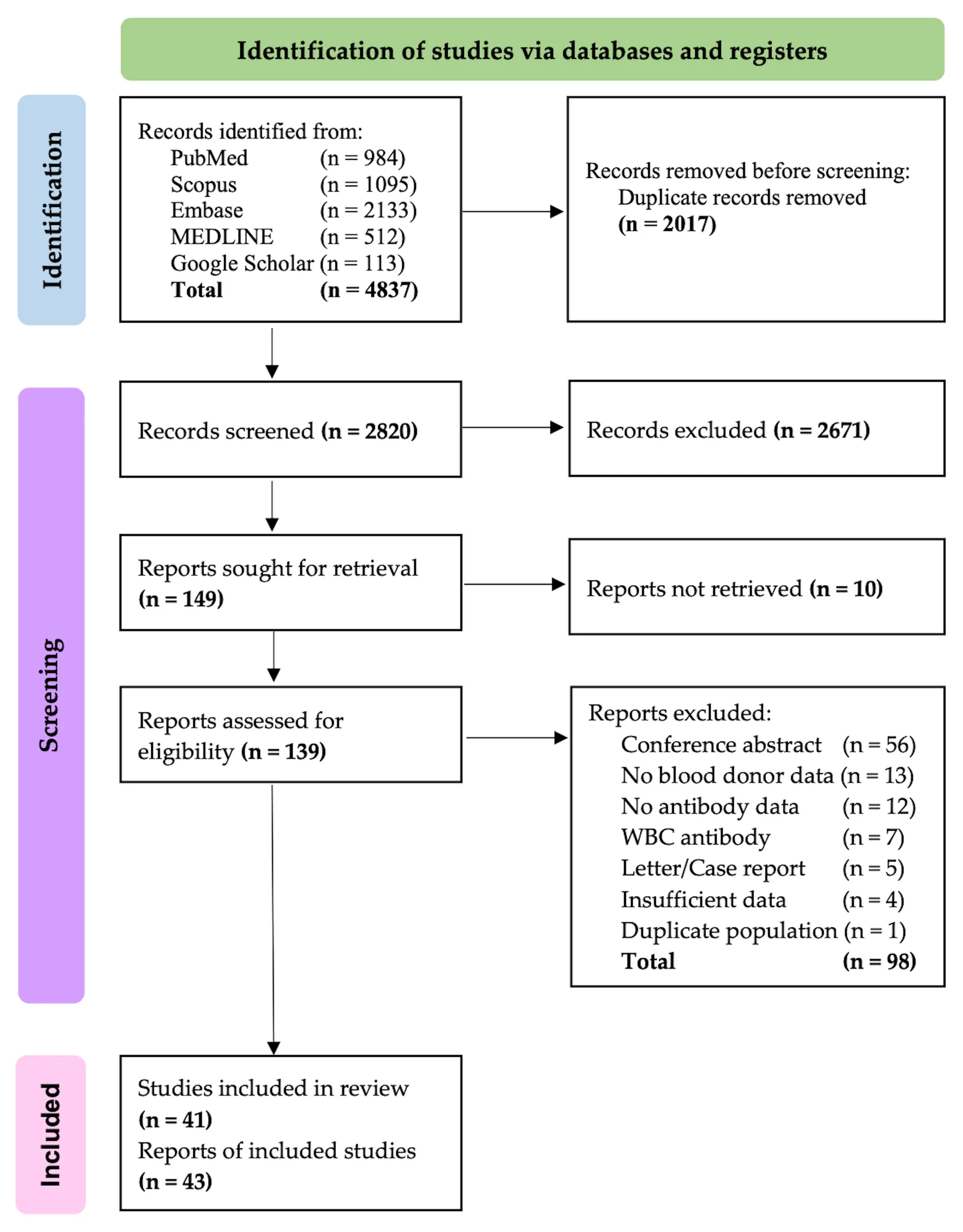
PRISMA flow diagram of study selection.

A total of 4837 records were identified through searches of five databases, including PubMed (*n* = 984), Scopus (*n* = 1095), Embase (*n* = 2133), MEDLINE (*n* = 512), and Google Scholar (*n* = 113). After removal of 2017 duplicate records, 2820 records remained for title and abstract screening. Of these, 2671 records were excluded based on irrelevance to the review objectives. A total of 149 records were sought for full-text retrieval, of which 10 reports could not be retrieved. Detailed retrieval attempts and reasons for non-retrieval are provided in Supplementary Table S3. The remaining 139 full-text articles were assessed for eligibility. Following the full-text evaluation, 98 articles were excluded for predefined reasons, including conference abstracts, lack of blood donor data, absence of antibody-related outcomes, focus on white blood cell antibodies, letters or case reports, insufficient data for analysis, and duplicate donor populations. Finally, 41 studies [9,10,11,12,16,21,22,23,24,25,26,27,28,29,30,31,32,33,34,35,36,37,38,39,40,41,42,43,44,45,46,47,48,49,50,51,52,53,54,55,56] met the inclusion criteria and were included in the systematic review. Two studies [30,46] reported results separately for distinct subgroups, resulting in a total of 43 reports included in the final meta-analysis. The study selection process is illustrated in Figure 1.

### 3.2. Study Characteristics

The included studies were published between 1965 and 2025, with the majority published during 2016–2025 (28 studies, 65.1%). Data were derived from 18 countries across multiple geographic regions, with most originating from Asia (26 studies, 60.5%). India contributed the largest number of studies (15 studies), followed by China (4 studies), France (3 studies), and the United States (3 studies). Regarding laboratory methods, most studies employed conventional tube testing (CTT) or column agglutination techniques (CAT), either alone or in combination (26 studies). Other methods included solid-phase red cell adherence assays (SPRCA), erythrocyte-magnetized technology (EMT), automated Groupamatic systems, and various combined testing approaches.

In total, 3,561,088 blood donors were screened for RBC antibodies. Sample size per study ranged from 75 to 661,511 donors and were broadly comparable across Asia (34.7%), Europe (32.9%), and the Americas (32.1%). France contributed the single largest donor population (1,142,891 donors), followed by the US (858,715 donors), and India (701,112 donors). Overall, the donor population was predominantly male (81.4%), while female donors accounted for 18.6% of screened donors. Antibody screening detection was positive in 16,443 donors. Notably, among donors with positive antibody screening results, a higher proportion were female (59.2%) compared with male donors (40.8%).

Data on ABO blood group distribution among screened donors were reported in a subset of studies. Blood group O was the most common (44.7%), followed by group A (36.1%), group B (14.2%), and group AB (5.1%). A similar distribution was observed among donors with antibody screening positivity, with group O accounting for 43.4%, followed by group A (35.2%), group B (15.2%), and group AB (6.2%). Detailed characteristics of the included studies are summarized in Table 1 and Supplementary Table S4.

### 3.3. Quality Assessment of Included Studies

Most included studies demonstrated high methodological quality. Of the 41 included studies, 31 studies (75.6%) were rated as high quality, while 10 studies (24.4%) were rated as moderate quality. No studies were classified as low quality. Most studies adequately defined the target population, employed appropriate sampling frames, and used valid and reliable methods for antibody screening and outcome measurement. Consequently, all included studies were considered to provide sufficient methodological rigor to contribute meaningfully to the pooled analyses. Detailed results of the quality assessment for each study are presented in Supplementary Table S5.

### 3.4. Overall Prevalence of Antibody Screening-Positive Donors

**Figure 2:**
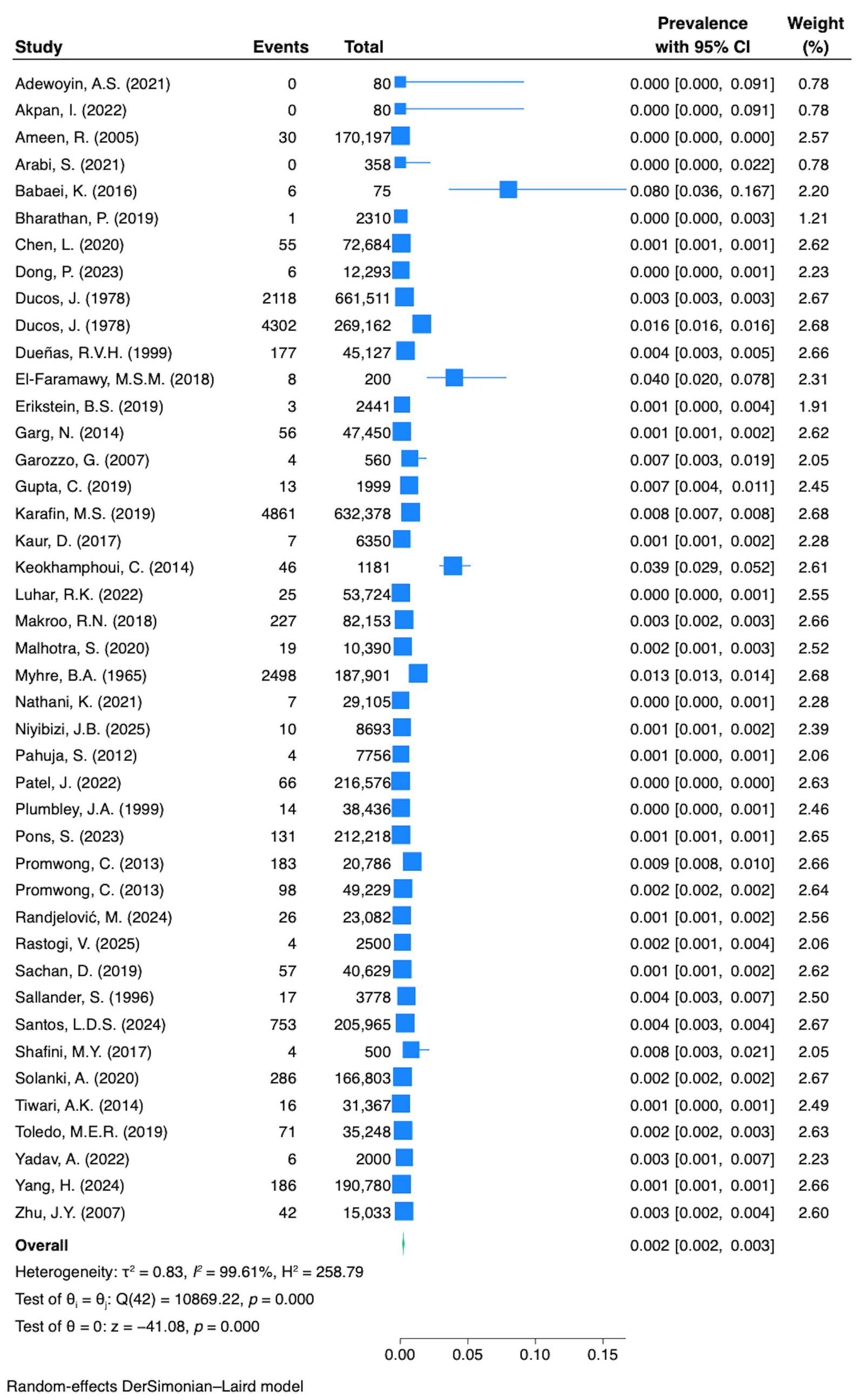
Forest plot of the prevalence of antibody screening positivity among blood donors. Each square represents the prevalence estimate from an individual study [9,10,11,12,16,21,22,23,24,25,26,27,28,29,30,31,32,33,34,35,36,37,38,39,40,41,42,43,44,45,46,47,48,49,50,51,52,53,54,55,56], with horizontal lines indicating 95% confidence intervals and square size reflecting study weight. The pooled prevalence estimate (diamond) was derived using a random-effects DerSimonian–Laird model with logit-transformed proportions. Heterogeneity was assessed using the *I*^2^ statistic.

Across the included studies, the prevalence of RBC antibody screening–positive donors varied widely. Using a random-effects meta-analysis, the pooled prevalence was 0.2% (95% CI 0.2–0.3%), with a 95% prediction interval of 0.0 to 1.4%. Individual study estimates ranged from 0.0% to 8.0%. Considerable between-study heterogeneity was observed (*I*^2^ = 99.61%) (Figure 2).

**Figure 3:**
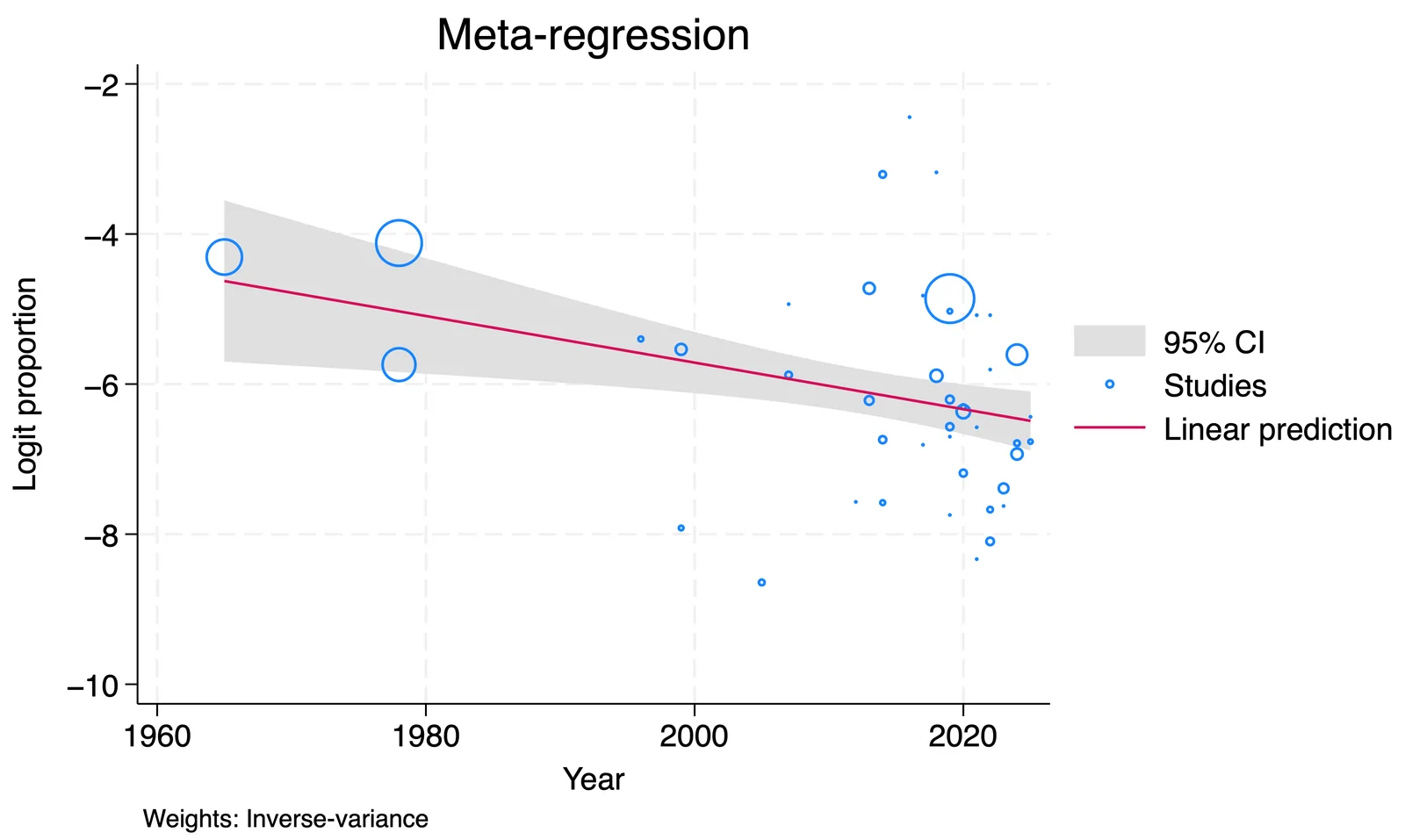
Bubble plot of meta-regression analysis examining the association between year of publication and the prevalence of antibody screening–positive blood donors. Each circle represents an individual study, with circle size proportional to inverse-variance weight. The solid line indicates the fitted linear regression, and the shaded area represents the 95% confidence interval.

Meta-regression analysis demonstrated an inverse association between publication year and the reported prevalence of antibody-positive donors (coefficient = −0.031 per year, 95% CI −0.053 to −0.010, *p* = 0.005) (Figure 3). This finding indicates that more recent studies tended to report lower prevalence estimates. However, substantial residual heterogeneity remained after accounting for publication year (*I*^2^ = 99.52%), suggesting that publication year did not explain the observed between-study variability.

### 3.5. Sex-Specific Prevalence of Antibody Screening-Positive Donors

**Figure 4:**
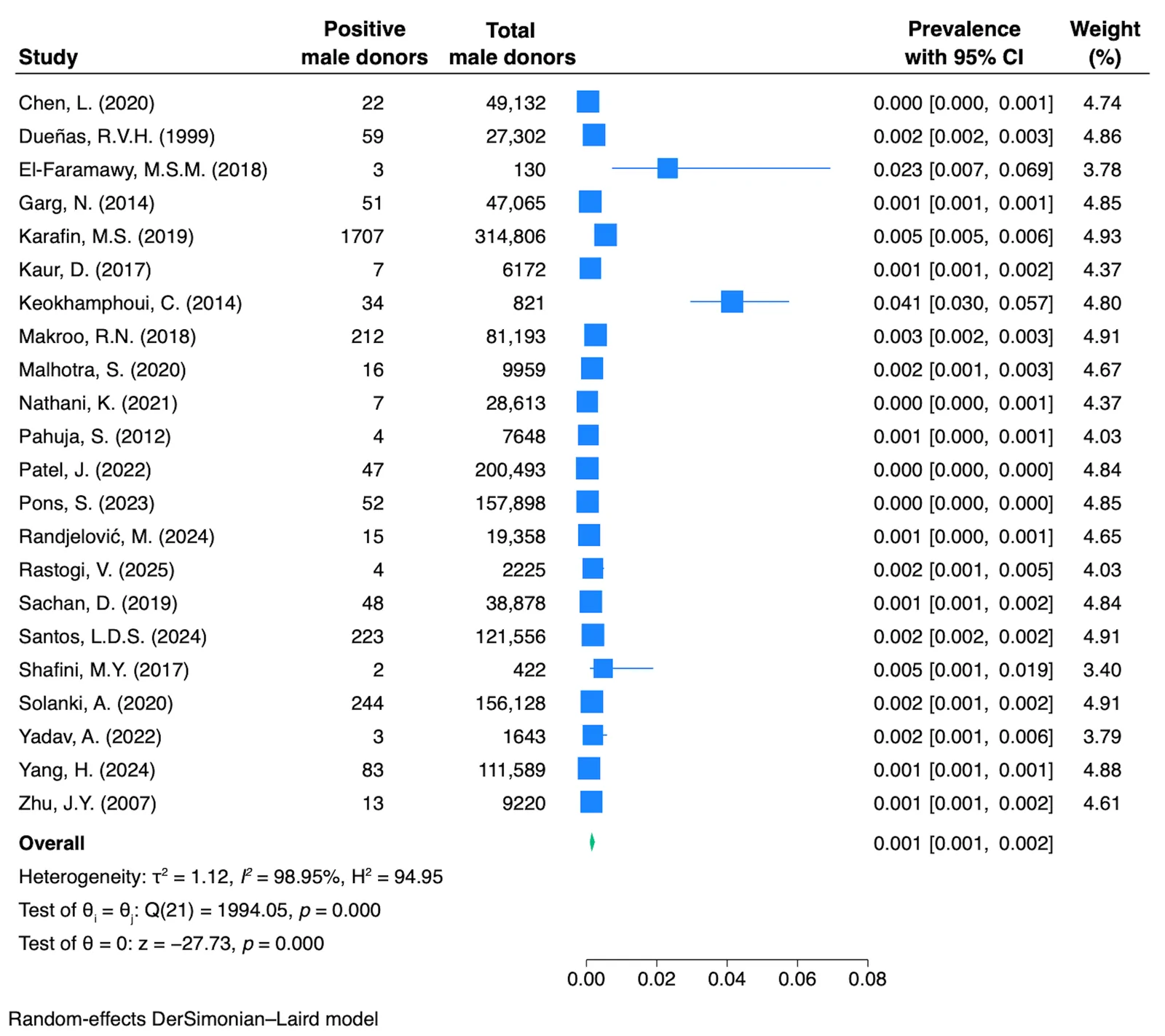
Forest plot showing the prevalence of antibody screening–positive male blood donors. Individual study estimates are presented as prevalence (%) with 95% confidence intervals, with square sizes proportional to study weights [9,10,11,12,16,21,28,31,35,36,37,39,41,43,44,47,48,50,51,54,55,56]. The pooled prevalence estimate (diamond) was calculated using a random-effects DerSimonian–Laird model with logit-transformed proportions.

**Figure 5:**
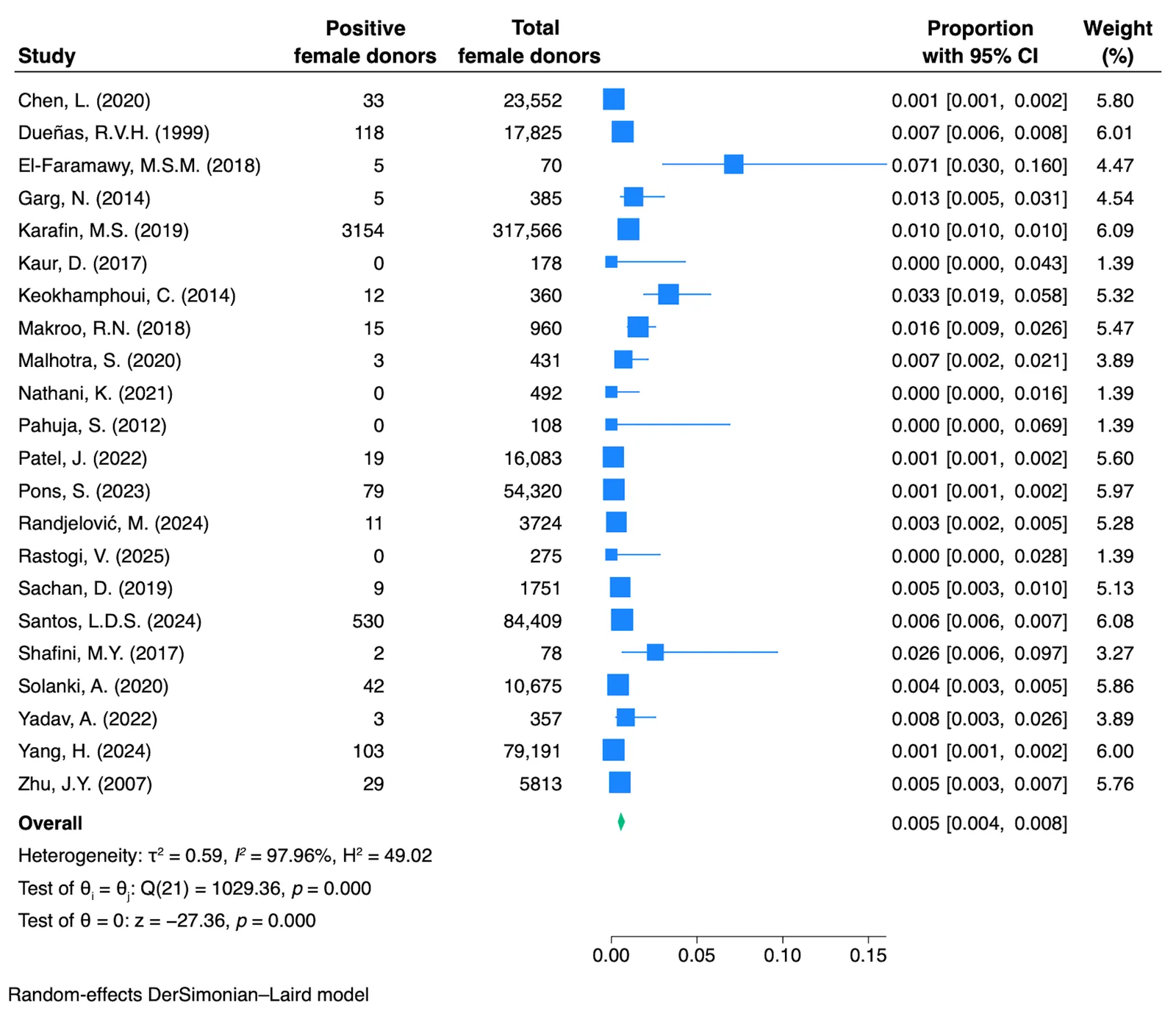
Forest plot showing the prevalence of antibody screening–positive female blood donors. Individual study estimates are presented as prevalence (%) with 95% confidence intervals, with square sizes proportional to study weights [9,10,11,12,16,21,28,31,35,36,37,39,41,43,44,47,48,50,51,54,55,56]. The pooled prevalence estimate (diamond) was calculated using a random-effects DerSimonian–Laird model with logit-transformed proportions.

Sex-specific prevalence data were available from 22 reports for both male and female donors. Among 1,392,251 male donors, the pooled prevalence of antibody screening-positive donors was 0.10% (95% CI 0.10–0.20%). Substantial heterogeneity was observed across studies (*I*^2^ = 98.95%) (Figure 4). In contrast, among 618,603 female donors, the pooled prevalence of antibody screening-positive donors was markedly higher at 0.50% (95% CI 0.40–0.80%), with similarly high between-study heterogeneity (*I*^2^ = 97.96%) (Figure 5). Across studies reporting sex-disaggregated data, antibody screening-positive donors were consistently more frequent in female donors than in male donors, despite males comprising the majority of screened donors.

### 3.6. Subgroup Analyses of Prevalence of Antibody Screening-Positive Donors

Given the high between-study heterogeneity observed in the overall analysis, subgroup analyses were performed to explore potential sources of variability. When stratified by antibody detection method, marked differences in pooled prevalence estimates were observed. Studies using Automated Groupamatic system reported a pooled prevalence of 0.7% (95% CI 0.1–3.4%). Studies employing conventional tube testing (CTT) showed a pooled prevalence of 0.8% (95% CI 0.4–1.8%), whereas those using column agglutination techniques (CAT) reported a lower pooled prevalence of 0.2% (95% CI 0.1–0.2%). Studies applying combined CTT and CAT methods yielded the pooled prevalence of 0.1% (95% CI 0.1–0.1%). Lower pooled prevalence estimates were also observed in studies using solid-phase red cell adherence assays (SPRCA) (0.3%, 95% CI 0.1–0.6%), EM technology (0.1%, 95% CI 0.0–0.3%), and microplate-based methods combined with CTT or CAT (0.1%, 95% CI 0.1–0.2%). The test for subgroup differences by detection method was statistically significant (*p* < 0.001), indicating that laboratory methodology contributed substantially to between-study heterogeneity (Supplementary Figure S1).

In contrast, subgroup analyses by geographic region revealed more modest variation in pooled prevalence estimates. The highest pooled prevalence was observed in Africa (0.7%, 95% CI 0.1–7.0%). The pooled prevalence of the Americas, Europe, and Asia were 0.3% (95% CI 0.2–0.5%), 0.3% (95% CI 0.1–0.7%), and 0.2% (95% CI 0.1–0.3%), respectively. The test for subgroup differences by geographic region yielded *p* = 0.117 and did not clearly distinguish the regional estimates from one another (Supplementary Figure S2). However, the wide confidence intervals, particularly for Africa, remained compatible with potentially meaningful regional variation, and substantial heterogeneity persisted within regions.

Subgroup analysis by publication period was conducted to examine temporal variation in reported RBC antibody prevalence. The pooled prevalence was 0.40% (95% CI 0.20–0.80%) among six reports published during 1965–1999, 0.20% (95% CI 0.1–0.6%) among nine reports published during 2000–2015, and 0.2% (95% CI 0.1–0.3%) among 28 reports published during 2016–2025. The test for differences among publication periods yielded *p* = 0.155, indicating that the period-specific estimates were not clearly distinguishable. Considerable variability among study estimates persisted within all three periods (*I*^2^ = 99.88%, 99.07%, and 99.28%, respectively) (Supplementary Figure S3).

### 3.7. Publication Bias and Small-Study Effects of RBC Antibody-Positive Donors

**Figure 6:**
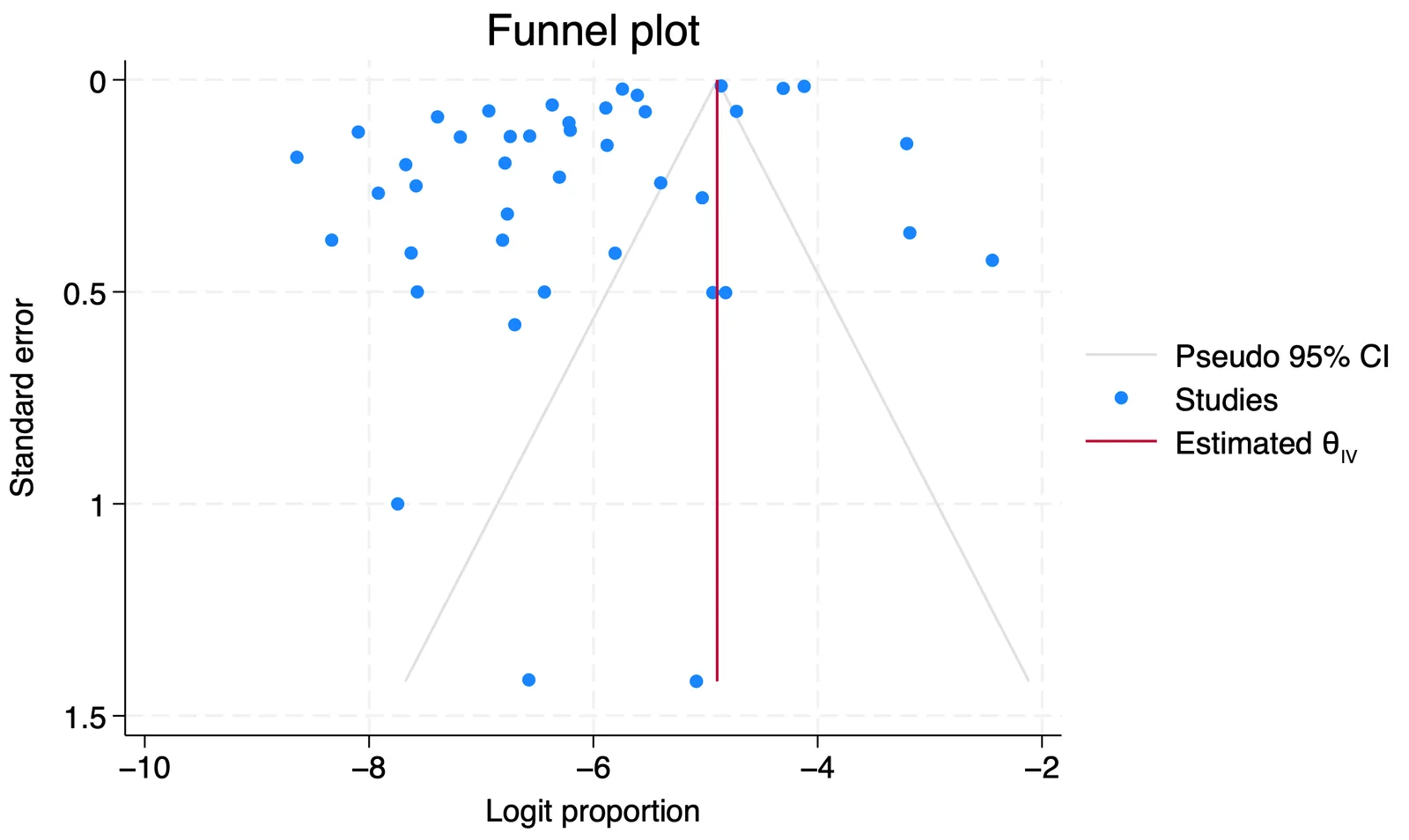
Funnel plot assessing publication bias for the prevalence of antibody screening–positive blood donors. Each point represents an individual study plotted against its standard error. The vertical line indicates the pooled effect estimate, and diagonal lines represent the pseudo 95% confidence limits.

Visual inspection of the funnel plot did not reveal marked asymmetry, suggesting no obvious evidence of publication bias (Figure 6). This observation was supported by the regression-based Egger test for small-study effects, which showed no statistically significant asymmetry (*p* = 0.598). Overall, these findings indicate no evidence of small-study effects or publication bias affecting the pooled prevalence estimates.

### 3.8. Association Between ABO Blood Group and RBC Antibody Detection

**Figure 7:**
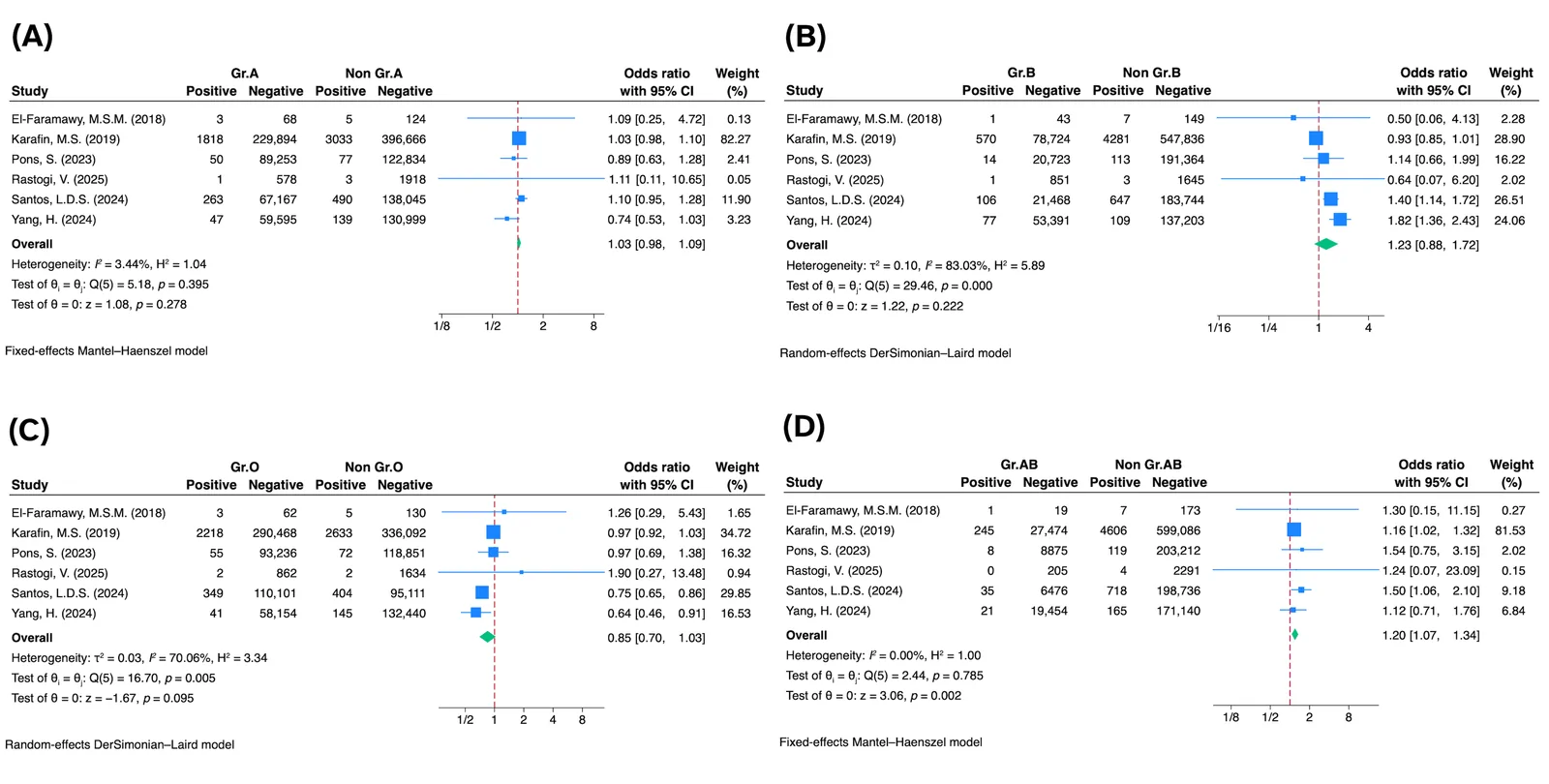
Forest plots showing the association between ABO blood group and the risk of positive antibody screening positive among blood donors. (**A**) Blood group A versus non-A, (**B**) blood group B versus non-B, (**C**) blood group O versus non-O, and (**D**) blood group AB versus non-AB. Pooled odds ratios (ORs) with 95% confidence intervals are shown, with square sizes proportional to study weights and diamonds representing pooled estimates [12,16,21,35,50,55]. Fixed- or random-effects models were applied according to heterogeneity.

The association between ABO blood group and RBC antibody detection in blood donors was evaluated using pooled odds ratios (ORs) from six studies reporting ABO-stratified data (Figure 7). For blood group A, the pooled estimate was close to null (OR 1.03, 95% CI 0.98–1.09) (Figure 7A). Blood group B showed higher estimated odds, although the confidence interval was wide (OR 1.23, 95% CI 0.88–1.72) (Figure 7B). Blood group O showed lower estimated odds compared with non-group O donors (OR 0.85, 95% CI 0.70–1.03) (Figure 7C). In contrast, blood group AB was associated with higher estimated odds of RBC antibody detection compared with non-AB donors (OR 1.20, 95% CI 1.07–1.34) (Figure 7D).

### 3.9. Sensitivity Analysis and Publication Bias of ABO Blood Group and Risk of RBC Antibody Detection

**Figure 8:**
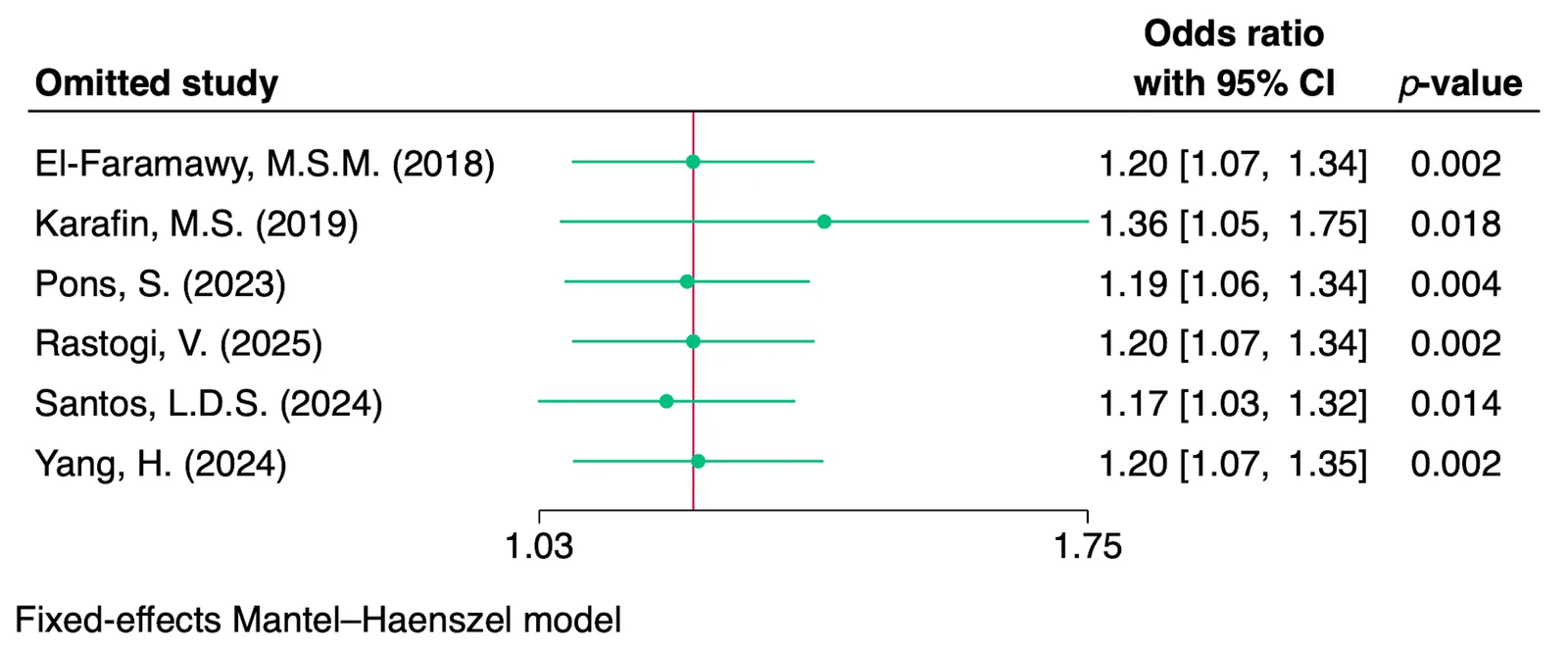
Leave-one-out sensitivity analysis of the association between blood group AB and antibody screening positivity in blood donors. Each point represents the pooled odds ratio (OR) with 95% confidence interval after sequential omission of one study [12,16,21,35,50,55].

**Figure 9:**
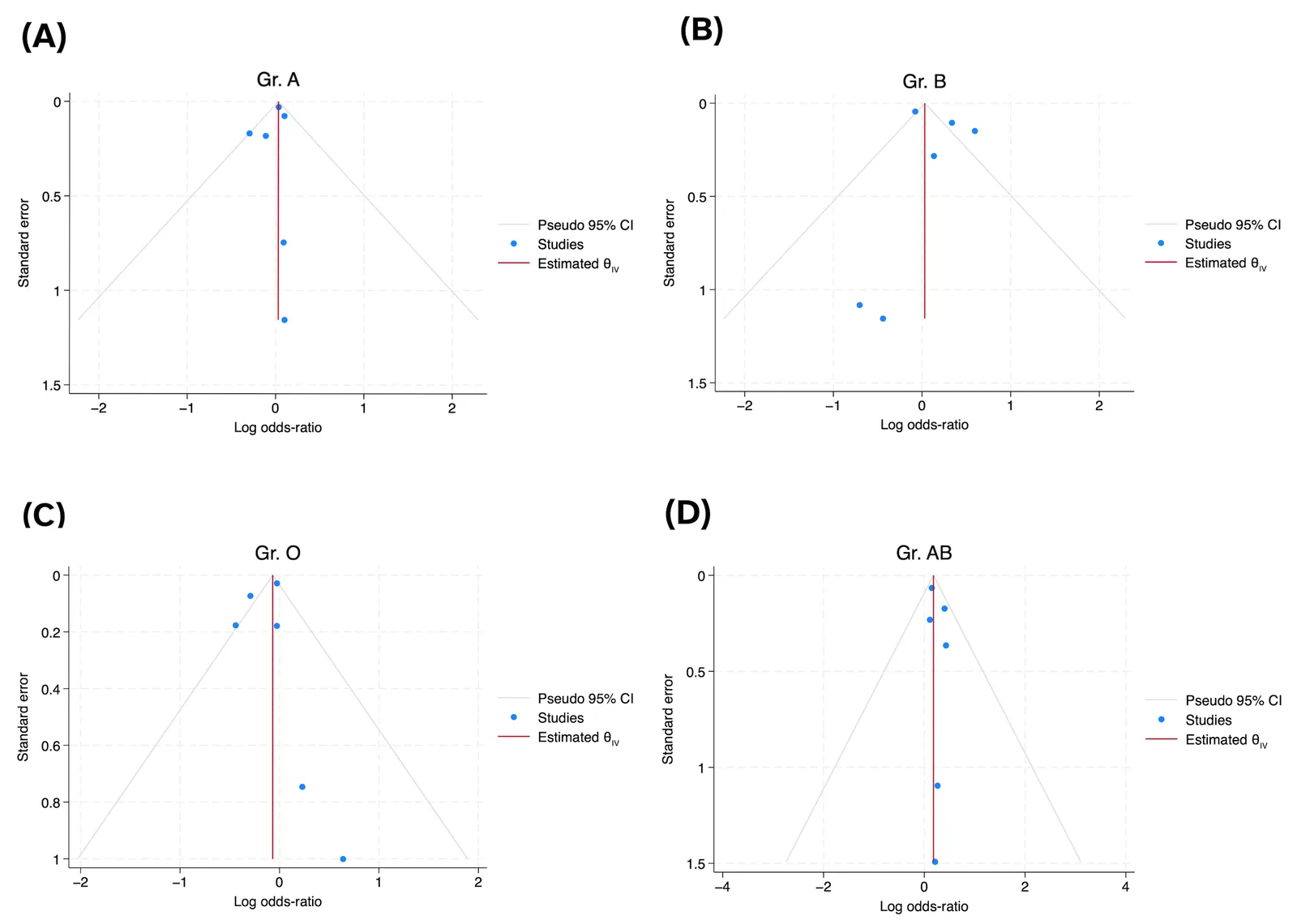
Funnel plots assessing publication bias for the association between ABO blood group and RBC antibody detection among blood donors. (**A**) Blood group A versus non-A, (**B**) blood group B versus non-B, (**C**) blood group O versus non-O, and (**D**) blood group AB versus non-AB.

Sensitivity analyses were conducted to assess the robustness of the pooled estimates for the association between group AB blood donors and RBC antibody detection. Leave-one-out analyses demonstrated that the pooled odds ratio for the remaining studies remained consistently significant after sequential omission of individual studies, with pooled ORs ranging from 1.17 to 1.37 and all corresponding confidence intervals excluding unity (Figure 8). These findings indicate that the observed association between blood group AB and increased antibody screening positivity was not driven by any single study. Visual inspection of funnel plots for blood groups A, B, O, and AB did not reveal substantial asymmetry (Figure 9). The distribution of study estimates appeared broadly symmetrical around the pooled effect size for each blood group, suggesting no clear evidence of publication bias or small-study effects.

### 3.10. Prevalence of RBC Alloantibody Specificities

The prevalence of RBC alloantibody specificities among antibody-positive blood donors was evaluated. Among immune-stimulated antibodies, anti-D was the most prevalent, with a pooled proportion of 18.1%, followed by anti-E (7.2%), anti-K (6.9%), and anti-Fy^a^ (5.5%). In addition, anti-Jk^a^ detection accounted for 4.1% of identified alloantibodies, while the combined anti-D + anti-C was observed in 4.5% of cases. Among naturally occurring alloantibodies, anti-Mi^a^ showed the highest pooled proportion (20.6%), followed by anti-M (14.1%). In the Lewis blood group system, both anti-Le^a^ (13.4%) and anti-Le^b^ (7.9%) were frequently reported, and the combined anti-Le^a^ + anti-Le^b^ was also commonly observed (8.9%). For the P1PK blood group system, anti-P1 accounted for 9.9% of identified alloantibodies. Details of the distribution of alloantibody frequency are presented in Table 2.

## 4. Discussion

This systematic review and meta-analysis provides a large global analysis of RBC antibody detection among blood donors, comprising more than 3.5 million donors from 18 countries. RBC antibody detection was uncommon overall, with a pooled prevalence of 0.2% (95% CI: 0.2–0.3%). This pooled estimate is consistent with previous donor studies in which antibody detection is typically reported in less than 1%of donors [38,43]. However, prevalence varied widely among individual studies, with considerable between-study heterogeneity. The 95% prediction interval of 0.0–1.4% was substantially wider than the confidence interval around the pooled mean, indicating that the underlying prevalence may differ markedly across donor populations and screening settings. Differences between initial screen reactivity and subsequently confirmed antibodies may also influence reported prevalence, as demonstrated in large donor series using different definitions and confirmatory workflows [11,16,39]. The prevalence observed among blood donors was markedly lower than that reported in transfusion recipients, in whom RBC alloimmunization has commonly been reported at approximately 0.5% to >10%, depending on underlying disease, transfusion exposure, and antigen-matching practices [57,58,59,60,61]. This difference is biologically plausible because transfusion recipients experience greater exposure to non-self RBC antigens, whereas most blood donors have fewer sensitizing exposures. The pooled prevalence was also higher among female than male donors. Pregnancy-related exposure to non-self RBC antigens provides a plausible explanation for part of this difference because fetomaternal hemorrhage is an established route of RBC alloimmunization [62]. However, pregnancy history, previous transfusion, age, and other donor-level characteristics were inconsistently reported across the included studies. Therefore, the observed difference cannot be attributed directly to pregnancy or interpreted as an adjusted sex effect. Accordingly, the pooled estimate should be interpreted as an international benchmark rather than as an estimate uniformly applicable to individual blood services.

Laboratory methodology was associated with variation in reported prevalence. Higher pooled estimates were observed with conventional tube testing and older automated platforms than with column agglutination technique and some combined approaches. These differences may reflect variation in analytical sensitivity, enhancement media, incubation conditions, interpretation procedures, and confirmatory algorithms. Comparative study have demonstrated that CAT and solid-phase methods may detect antibody reactivity patterns that differ from those identified by tube testing, particularly for weak or low-titer antibodies [63,64,65]. Automation and standardized interpretation can reduce operator variability and improve traceability [66]. However, detection method was closely related to publication period and study setting, limiting separation of methodological, temporal, and geographic effects. Temporal analyses should therefore be interpreted cautiously. Prevalence decreased numerically from 0.4% in studies published during 1965–1999 to 0.2% in both 2000–2015 and 2016–2025. Meta-regression demonstrated an inverse association between publication year and reported prevalence, but substantial residual heterogeneity remained and publication year did not explain the estimated between-study variance. Calendar time also coincided with major changes in screening platforms, reagent panels, donor-selection practices, information systems, definitions of screening reactivity, and confirmatory procedures [67,68,69]. The available evidence therefore cannot determine whether RBC antibody prevalence has truly declined over time. Earlier studies should instead be interpreted within their historical methodological context. Considerable variability also remained after subgroup and meta-regression analyses, indicating that laboratory method, geographic region, and publication period did not fully account for the differences among studies. Other plausible contributors include donor-selection and deferral policies, the proportion of first-time versus repeat donors, previous pregnancy or transfusion, and ethnic variation in RBC antigen frequencies [69,70]. Geographic region should not be interpreted as a proxy for race or ethnicity. Differences in reagent-cell sensitivity, thresholds for defining screening reactivity, antibody-identification panels, and confirmatory protocols may also have contributed to variation [63,64,65]. Because these characteristics were inconsistently reported, their independent effects could not be quantified.

The antibody-specificity analysis further demonstrated substantial heterogeneity in the immunologic profiles detected among blood donors. Among predominantly immune-stimulated antibodies, anti-D had the highest pooled proportion, followed by anti-E, anti-K, anti-Fy^a^, and anti-Jk^a^. The prominence of Rh and Kell antibodies is consistent with their established immunogenicity and clinical importance in transfusion and pregnancy [71,72]. Naturally occurring antibodies, including anti-M, anti-P1, anti-Le^a^, and anti-Le^b^, and anti-Mi^a^, were also frequently detected. Similar predominance of clinically important Rh and Kell specificities has been reported in transfused patient populations. A Brazilian hospital-based study reported anti-D, anti-E, and anti-K as the most frequent clinically significant antibodies among patients with irregular antibodies [73]. A study in β-thalassemia patients identified anti-K and anti-D as prominent specificities [74]. In an Indian tertiary-care cohort, anti-E and anti-D were the most frequent single alloantibodies, with anti-Jk^a^ also commonly observed [13]. Nevertheless, direct comparison with blood donors should be made cautiously because the patterns and intensity of antigen exposure differ substantially. Regional variation was particularly evident for anti-Mi^a^. Mi(a) is a low-frequency red cell antigen associated with the MNS glycophorin variants that are more prevalent in Southeast Asia and South China [75,76,77]. Previous studies from Thailand and Malaysia have similarly reported anti-Mi^a^ among frequently detected RBC antibodies [78,79,80]. The relatively high pooled proportion of anti-Mi^a^ in this meta-analysis likely reflects the concentration of contributing studies in populations in which Mi^a^-related glycophorin variants are more frequent. However, this estimate was based on only three studies and should interpreted cautiously.

The ABO-stratified analysis was based on six studies and should therefore be considered exploratory. Blood group AB was associated with higher estimated odds of RBC antibody detection compared with non-AB donors, whereas the estimate for blood group O suggested lower odds but its confidence interval included the null. Evidence from transfused patient populations has also been inconsistent. One study reported lower adjusted odds of RBC alloimmunization among individuals with blood groups A and O compared with group AB [81], whereas another found no association between ABO blood group and RBC immunization [82]. Variation at the ABO locus influences glycosyltransferase activity and has been associated with inflammatory and endothelial adhesion markers, providing a potential biological context for an association between ABO-related glycosylation and immune responsiveness [83,84,85]. However, this mechanism was not evaluated in the included studies, and direct evidence linking ABO phenotype to RBC alloantibody formation remains limited. Furthermore, the present analysis relied on aggregate, unadjusted data, and residual confounding by sex, pregnancy history, previous transfusion, ethnicity, donor-selection practices, and laboratory methodology cannot be excluded. Accordingly, the observed association does not establish causality and should not be used to support ABO-based donor exclusion, risk classification, or differential antibody-screening policies.

The clinical relevance of donor-derived RBC antibodies depends strongly on the component transfused. Exposure to donor antibodies is generally greater with whole blood and plasma-rich components, including plasma and platelet products, than with RBC concentrates prepared in additive solution [86]. Clinical significance also depends on antibody specificity, titer, thermal amplitude, immunoglobulin class, the volume of plasma transfused, and recipient antigen expression [87]. Passive transfer of donor antibodies has historically been associated with hemolytic complications, particularly when appreciable volumes of donor plasma are transfused [88]. By contrast, observational evidence suggests that the appropriately processed RBC concentrates containing donor alloantibodies may pose a relatively low clinical risk of the small residual plasma volume [89]. Such observations, however, do not establish an absence of risk, particularly in vulnerable recipients or when larger volumes of antibody-containing plasma are transfused. Current guidance and blood-service practices illustrate component-specific management of donations containing RBC antibodies. UK donation-testing guidance includes RBC antibody screening and requires completion and documentation of mandatory testing before components are released [90]. Canadian Blood Services permits appropriately labeled RBC concentrates containing donor antibodies for adult transfusion because of their low residual plasma volume, whereas plasma and platelet components are not produced from donations containing RBC alloantibodies [91]. The current European Directorate for the Quality of Medicines & HealthCare (EDQM) provides broader European standards for donation testing, component preparation, quality assurance, and hemovigilance [92]. These approaches emphasize that management should consider component type, residual plasma volume, antibody characteristics, processing practices, and hemovigilance evidence rather than donor antibody detection alone.

Donor-screening strategies also vary among blood services and should be evaluated within the local operational and regulatory contexts. Australian Red Cross Lifeblood evaluated an alternative strategy in which screening would be performed at the first donation and repeated after pregnancy, transfusion, or a donation interval exceeding two years. Under this strategy, 47 donors with clinically significant antibodies would have remained undetected. Although the alternative was considered more cost-effective, the additional residual risk was considered unacceptable in that setting [69]. The present meta-analysis evaluated antibody prevalence rather than the comparative effectiveness, cost-effectiveness, or clinical outcomes of universal versus selective screening and therefore cannot establish the optimal screening strategy for individual blood services. Decisions should instead consider local antibody prevalence, donor demographics, sensitizing exposures, component-processing practices, laboratory capacity, regulatory requirements, blood-supply implications, and hemovigilance data. Where universal screening is already established, the low prevalence observed in this review should not alone justify its discontinuation because prevalence does not directly quantify the clinical consequences of undetected antibodies.

This review has several strengths, including a registered protocol, a broad literature search, inclusion of more than 3.5 million blood donors, and evaluation of overall and sex-specific prevalence, laboratory methodology, geographic and temporal variation, ABO blood group associations, and antibody-specificity patterns. The use of subgroup analyses, meta-regression, leave-one-out sensitivity analysis, and a prediction interval also allowed the extent and potential sources of between-study variability to be examined. However, several limitations should be considered. First, the geographic distribution of included studies was uneven, with limited representation from low-resource and low-income regions. This may limit direct applicability of the findings to blood services with different donor demographics, laboratory infrastructure, and transfusion practices [93]. Donor-level characteristics were inconsistently reported and could not be quantitatively evaluated. Second, substantial between-study heterogeneity persisted despite subgroup and meta-regression analyses. The included studies covered a period of approximately six decades, during which antibody detection evolved from predominantly manual tube and enzyme-based methods to automated Groupamatic systems, column agglutination, solid-phase red cell adherence assays, and erythrocyte-magnetized technology. Concurrent changes in reagent-cell composition, enhancement media, incubation conditions, analytical sensitivity, definitions of screening positivity, and confirmatory identification algorithms may have influenced the reported prevalence [33,49,63,64,65,66,67,68]. Third, incomplete or inconsistent reporting of antibody identification may have resulted in misclassification and imprecision in pooled specificity estimates. Inadequate reporting of previous pregnancy and transfusion also limited adjustment for important determinants of immune-stimulated antibody formation [5]. Fourth, the ABO analysis included only six studies should therefore be considered exploratory. Finally, funnel plot symmetry and Egger regression did not provide clear evidence of small-study effects; however, selective reporting cannot be excluded [94].

## 5. Conclusions

This systematic review and meta-analysis demonstrates that RBC antibody detection among blood donors is uncommon worldwide but varies considerably across donor populations, laboratory methods, and study periods. Prevalence was higher among female donors. However, the available aggregate data did not permit direct attribution of this difference to pregnancy or other sensitizing exposures. More recent studies tended to report lower prevalence estimates, although this temporal pattern could not be separated from concurrent changes in screening platforms, reagent panels, donor selection, outcome definitions, and confirmatory procedures. Predominantly immune-stimulated antibodies within the Rh and Kell systems were frequently reported, while regionally enriched specificities, particularly anti-Mi^a^, highlighted the importance of population-specific antigen distributions. Given the considerable between-study variability, the pooled estimate should be interpreted as an international benchmark rather than as a uniform prevalence applicable to individual blood services. These findings support context-specific approaches to donor antibody screening with confirmatory testing, component management, and hemovigilance, guided by local prevalence, laboratory capacity, regulatory requirements, and transfusion practices.

## Figures and Tables

**Table 1:** Summary characteristics of included studies.

Characteristics	Study, *n* (%)	Donor, *n* (%)
Publication year		
Before 2000	6 (14.0%)	1,205,915 (33.9%)
2000–2015	9 (20.9%)	343,559 (9.6%)
2016–2025	28 (65.1%)	2,011,614 (56.5%)
Country		
Asia	26 (60.5%)	1,234,228 (34.7%)
China	4	290,790
India	15	701,112
Iran	2	433
Kuwait	1	170,197
Laos	1	1181
Malaysia	1	500
Thailand	2	70,015
Europe	7 (16.3%)	1,172,752 (32.9%)
France	3	1,142,891
Italy	1	560
Norway	1	2441
Serbia	1	23,082
Sweden	1	3778
Americas	6 (13.9%)	1,145,055 (32.1%)
Brazil	1	205,965
Colombia	2	80,375
USA	3	858,715
Africa	4 (9.3%)	9053 (0.3%)
Egypt	1	200
Nigeria	2	160
Rwanda	1	8693
Antibody detection method		
CTT	10 (23.3%)	425,985 (12.0%)
CAT	12 (27.9%)	194,531 (5.5%)
CTT and CAT	4 (9.3%)	302,959 (8.5%)
SPRCA	5 (11.6%)	504,674 (14.2%)
EMT	3 (7.0%)	392,072 (11.0%)
Microplate and CTT/CAT	3 (7.0%)	73,811 (2.1%)
Automated Groupamatic	2 (4.7%)	930,673 (26.1%)
SPRCA and EM technology	1 (2.3%)	53,724 (1.5%)
SPRCA, CTT or CAT	1 (2.3%)	632,378 (17.8%)
N/A	2 (4.7%)	50,281 (1.4%)
Male: Female of total donors		
Male	29 (67.4%)	1,476,211 (81.4%)
Female		336,837 (18.6%)
Male: Female of unexpected antibody-positive donors		
Male	25 (58.1%)	2918 (40.8%)
Female		4225 (59.2%)
ABO blood group of total donors		
A	10 (23.3%)	449,457 (36.1%)
B		176,920 (14.2%)
O		556,922 (44.7%)
AB		63,089 (5.1%)
ABO blood group of unexpected antibody-positive donors		
A	17 (39.5%)	2351 (35.2%)
B		1014 (15.2%)
O		2903 (43.4%)
AB		417 (6.2%)

Abbreviations: CAT, column agglutination technique; CTT, conventional tube testing; EMT, erythrocyte-magnetized technology; N/A, not available; SPRCA, solid-phase red cell adherence assay.

**Table 2:** Prevalence of RBC alloantibody specificities among antibody-positive blood donors.

Blood Group System	Alloantibody Specificity	Studies, *n*	Screened Donors, *n*	Alloantibody- Positive Donors, *n*	Donors with Specific Alloantibody, *n*	Pooled Proportion, % (95% CI)
Rh ^†^	D	23	3,047,281	10,480	2767	18.1 (15.2–21.4)
	C	13	2,121,991	7865	396	4.5 (2.3–8.5)
	E	21	3,020,841	10,622	1363	7.2 (4.0–12.9)
	c	15	2,649,097	9887	302	3.6 (2.1–6.2)
	e	4	1,510,540	7043	34	1.2 (0.4–3.9)
	C^w^	6	1,150,399	2570	52	3.7 (1.6–8.7)
	D + C	7	1,226,292	4696	337	4.5 (2.2–8.9)
	D + E	5	1,333,383	4882	20	0.4 (0.3–0.7)
MNS ^§^	M	28	2,321,863	5818	292	14.1 (8.0–23.6)
	N	12	1,717,227	3039	60	3.7 (1.7–7.9)
	S	9	1,712,279	5660	126	3.0 (2.0–4.5)
	s	3	296,951	302	4	1.8 (0.7–4.6)
	Mi^a^	3	70,515	285	35	20.6 (5.3–54.8)
P1PK ^‡^	P1	12	1,688,926	5153	921	9.9 (6.7–14.4)
Lewis ^‡^	Le^a^	22	2,107,442	5522	643	13.4 (9.1–19.1)
	Le^b^	16	1,966,718	5431	267	7.9 (4.5–13.7)
	Le^a^ + Le^b^	6	262,594	311	22	8.9 (5.1–15.2)
Kell ^†^	K	17	2,607,332	9972	1148	6.9 (3.0–15.1)
	k	1	45,127	177	1	0.6 (0.1–3.9)
	Kp^a^	2	265,942	136	2	1.9 (0.5–7.3)
	Kp^b^	1	166,803	256	7	2.7 (1.3–5.6)
Kidd ^†^	Jk^a^	6	1,151,882	5398	223	4.1 (3.6–4.7)
	Jk^b^	5	1,263,223	5353	30	1.4 (0.3–5.8)
Duffy ^†^	Fy^a^	6	1,130,070	5297	289	5.5 (4.9–6.2)
	Fy^b^	2	799,181	5117	25	1.1 (0.1–12.2)
Lutheran ^§^	Lu^a^	0	0	0	0	-
	Lu^b^	1	166,803	256	5	2.0 (0.8–4.6)

Abbreviations: CI, confidence interval; RBC, red blood cell; Rh, Rhesus blood group system; MNS, MNS blood group system. ^†^ Predominantly immune-stimulated antibodies; ^‡^ usually naturally occurring antibodies; ^§^ antibodies of mixed origin, which may occur naturally or after transfusion or pregnancy. Within the MNS system, anti-M and anti-N are usually naturally occurring, anti-S and anti-s are predominantly immune, and anti-Mi^a^ may be either; anti-Lu^a^ may be naturally occurring or immune, whereas anti-Lu^b^ is more commonly immune. Pooled proportions were calculated among antibody-positive blood donors in studies reporting each specific alloantibody. A dash indicates that no eligible study reported the corresponding specificity.

## Data Availability

All data generated or analyzed during this study are included in this published article and its Supplementary Materials.

## Supplementary Materials

The following supporting information can be downloaded at: https://www.mdpi.com/article/10.3390/diseases14080298/s1, Figure S1: Forest plot of subgroup analyses of the prevalence of RBC antibody detection among blood donors according to antibody screening method; Figure S2: Forest plot of subgroup analyses of the prevalence of RBC antibody detection among blood donors according to geographic region; Figure S3: Forest plot of subgroup analyses of the prevalence of RBC antibody detection among blood donors according to publication period; Table S1: PRISMA 2020 Checklist; Table S2: Search strategies; Table S3: Characteristics of reports that remained unavailable in full text after retrieval attempts; Table S4: Characteristics and antibody detection outcomes of included studies; Table S5: Quality assessment of included studies.

## Abbreviations

The following abbreviations are used in this manuscript:

ABO	ABO blood group system
CAT	Column agglutination technique
CI	Confidence interval
CTT	Conventional tube testing
EDQM	European Directorate for the Quality of Medicines & HealthCare
EMT	Erythrocyte-magnetized technology
IgG	Immunoglobulin G
IgM	Immunoglobulin M
JBI	Joanna Briggs Institute
MNS	MNS blood group system
OR	Odds ratio
PRISMA	Preferred Reporting Items for Systematic Reviews and Meta-Analyses
PROSPERO	International Prospective Register of Systematic Reviews
RBC	Red blood cell
Rh	Rhesus blood group system
SPRCA	Solid-phase red cell adherence assay
WBC	White blood cell
