# Supplementary material for: Global Prevalence and Specificity of Red Blood Cell Antibodies in Blood Donors: A Systematic Review and Meta-Analysis
Source: Diseases. 2026 Aug 18;14(8):298. doi: 10.3390/diseases14080298 (PMC13512737; doi:10.3390/diseases14080298)
Supplement: Supplementary file 1 [file diseases-14-00298-s001.zip › Supplementary Figures S1–S3.pdf]

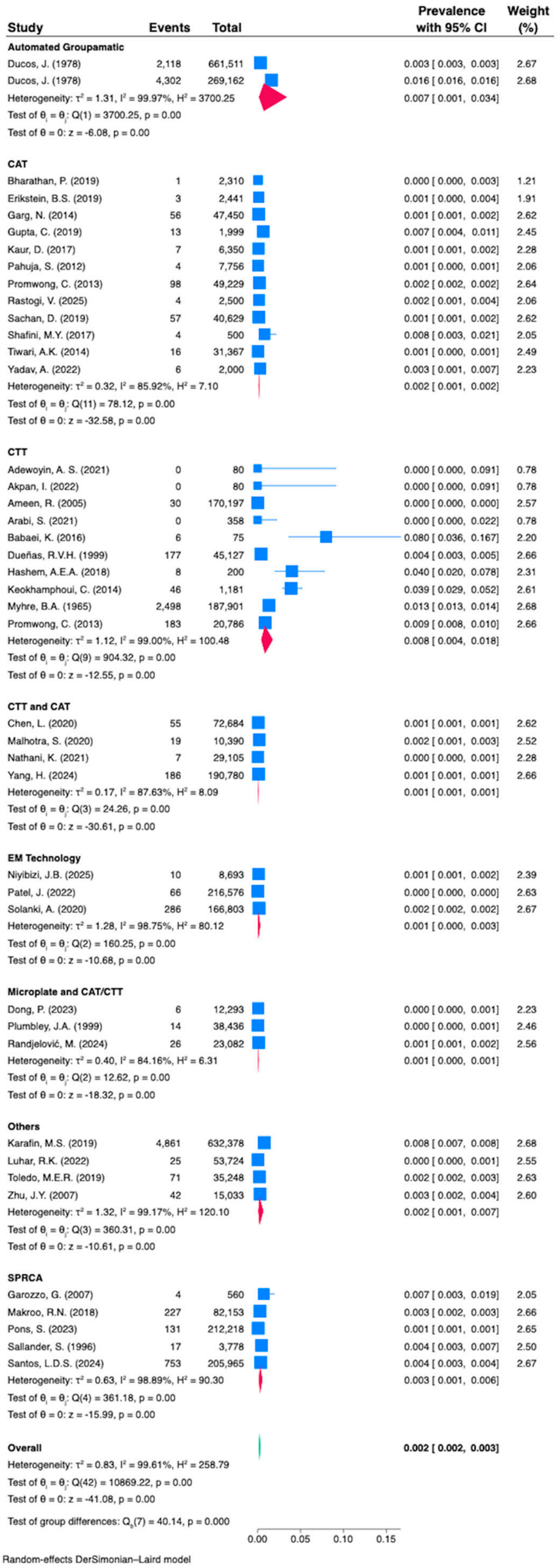

Figure S1. Forest plot of subgroup analyses of the prevalence of RBC antibody detection among blood donors according to antibody screening method. Studies were stratified by antibody screening methodology, including automated Groupamatic systems, column agglutination technique (CAT), conventional tube testing (CTT), combined CTT and CAT, erythrocyte magnetized technology (EMT), microplate-based methods combined with CAT, other combined methods, and solid-phase red cell adherence assay (SPRCA).

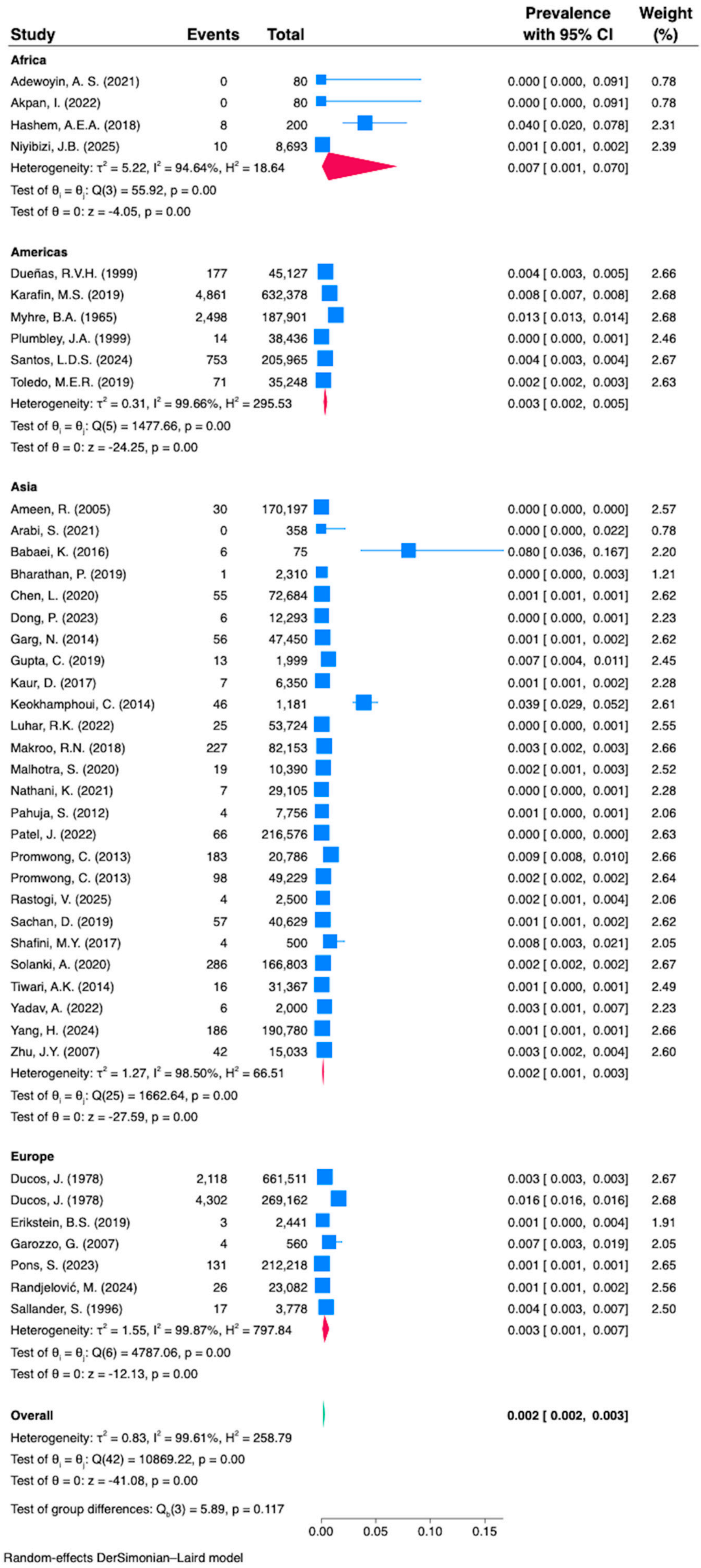

Figure S2. Forest plot of subgroup analyses of the prevalence of RBC antibody detection among blood donors according to geographic region. Studies were stratified into Africa, the Americas, Asia, and Europe.

Study	Events	Total	Prevalence with 95% CI	Weight (%)
**1965-1999**				
Ducos, J. (1978)	2,118	661,511	0.003 [0.003, 0.003]	2.67
Ducos, J. (1978)	4,302	269,162	0.016 [0.016, 0.016]	2.68
Dueñas, R.V.H. (1999)	177	45,127	0.004 [0.003, 0.005]	2.66
Myhre, B.A. (1965)	2,498	187,901	0.013 [0.013, 0.014]	2.68
Plumbley, J.A. (1999)	14	38,436	0.000 [0.000, 0.001]	2.46
Sallander, S. (1996)	17	3,778	0.004 [0.003, 0.007]	2.50
Heterogeneity: $\tau^2 = 0.73$, $I^2 = 99.88\%$, $H^2 = 850.46$			0.004 [0.002, 0.008]	
Test of $\theta_i = \theta_j$: Q(5) = 4252.28, p = 0.00				
Test of $\theta = 0$: z = -15.50, p = 0.00				
**2000-2015**				
Ameen, R. (2005)	30	170,197	0.000 [0.000, 0.000]	2.57
Garg, N. (2014)	56	47,450	0.001 [0.001, 0.002]	2.62
Garozzo, G. (2007)	4	560	0.007 [0.003, 0.019]	2.05
Keokhamphoui, C. (2014)	46	1,181	0.039 [0.029, 0.052]	2.61
Pahuja, S. (2012)	4	7,756	0.001 [0.000, 0.001]	2.06
Promwong, C. (2013)	183	20,786	0.009 [0.008, 0.010]	2.66
Promwong, C. (2013)	98	49,229	0.002 [0.002, 0.002]	2.64
Tiwari, A.K. (2014)	16	31,367	0.001 [0.000, 0.001]	2.49
Zhu, J.Y. (2007)	42	15,033	0.003 [0.002, 0.004]	2.60
Heterogeneity: $\tau^2 = 2.32$, $I^2 = 99.07\%$, $H^2 = 107.98$			0.002 [0.001, 0.006]	
Test of $\theta_i = \theta_j$: Q(8) = 863.80, p = 0.00				
Test of $\theta = 0$: z = -11.95, p = 0.00				
**2016-2025**				
Adewoyin, A. S. (2021)	0	80	0.000 [0.000, 0.091]	0.78
Akpan, I. (2022)	0	80	0.000 [0.000, 0.091]	0.78
Arabi, S. (2021)	0	358	0.000 [0.000, 0.022]	0.78
Babaei, K. (2016)	6	75	0.080 [0.036, 0.167]	2.20
Bharathan, P. (2019)	1	2,310	0.000 0.000, 0.003]	1.21
Chen, L. (2020)	55	72,684	0.001 [0.001, 0.001]	2.62
Dong, P. (2023)	6	12,293	0.000 [0.000, 0.001]	2.23
Hashem, A.E.A. (2018)	8	200	0.040 [0.020, 0.078]	2.31
Erikstein, B.S. (2019)	3	2,441	0.001 [0.000, 0.004]	1.91
Gupta, C. (2019)	13	1,999	0.007 [0.004, 0.011]	2.45
Karafin, M.S. (2019)	4,861	632,378	0.008 [0.007, 0.008]	2.68
Kaur, D. (2017)	7	6,350	0.001 [0.001, 0.002]	2.28
Luhar, R.K. (2022)	25	53,724	0.000 [0.000, 0.001]	2.55
Makroo, R.N. (2018)	227	82,153	0.003 [0.002, 0.003]	2.66
Malhotra, S. (2020)	19	10,390	0.002 [0.001, 0.003]	2.52
Nathani, K. (2021)	7	29,105	0.000 [0.000, 0.001]	2.28
Niyibizi, J.B. (2025)	10	8,693	0.001 [0.001, 0.002]	2.39
Patel, J. (2022)	66	216,576	0.000 [0.000, 0.000]	2.63
Pons, S. (2023)	131	212,218	0.001 [0.001, 0.001]	2.65
Randjelović, M. (2024)	26	23,082	0.001 [0.001, 0.002]	2.56
Rastogi, V. (2025)	4	2,500	0.002 [0.001, 0.004]	2.06
Sachan, D. (2019)	57	40,629	0.001 [0.001, 0.002]	2.62
Santos, L.D.S. (2024)	753	205,965	0.004 [0.003, 0.004]	2.67
Shafini, M.Y. (2017)	4	500	0.008 [0.003, 0.021]	2.05
Solanki, A. (2020)	286	166,803	0.002 [0.002, 0.002]	2.67
Toledo, M.E.R. (2019)	71	35,248	0.002 [0.002, 0.003]	2.63
Yadav, A. (2022)	6	2,000	0.003 [0.001, 0.007]	2.23
Yang, H. (2024)	186	190,780	0.001 [0.001, 0.001]	2.66
Heterogeneity: $\tau^2 = 1.15$, $I^2 = 99.28\%$, $H^2 = 139.73$			0.002 [0.001, 0.003]	
Test of $\theta_i = \theta_j$: Q(27) = 3772.83, p = 0.00				
Test of $\theta = 0$: z = -28.63, p = 0.00				
**Overall**			0.002 [0.002, 0.003]	
Heterogeneity: $\tau^2 = 0.83$, $I^2 = 99.61\%$, $H^2 = 258.79$				
Test of $\theta_i = \theta_j$: Q(42) = 10869.22, p = 0.000				
Test of $\theta = 0$: z = -41.08, p = 0.000				
Test of group differences: $Q_b(2) = 3.72$, p = 0.16				

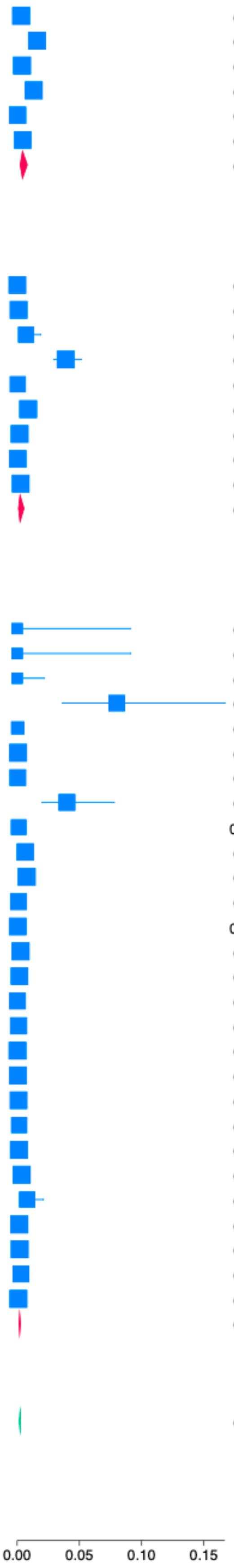

Random-effects DerSimonian–Laird model

Figure S3. Forest plot of subgroup analyses of the prevalence of RBC antibody detection among blood donors according to publication period. Studies were stratified into 3 periods; 1965-1999, 2000-2015, 2016-2025.
