# Supplementary material for: Global Prevalence and Specificity of Red Blood Cell Antibodies in Blood Donors: A Systematic Review and Meta-Analysis
Source: Diseases. 2026 Aug 18;14(8):298. doi: 10.3390/diseases14080298 (PMC13512737; doi:10.3390/diseases14080298)
Supplement: Supplementary file 1 [file diseases-14-00298-s001.zip › Table S2. Search results.pdf]

Table S2. Search strategies

Database	Search strategy	Date	Search results
Pubmed	("blood donors"[MeSH Terms] OR "blood donor*"[Title/Abstract] OR "blood donation"[Title/Abstract] OR "donor screening"[Title/Abstract] OR "voluntary donor*"[Title/Abstract] OR "replacement donor*"[Title/Abstract] OR "repeat donor*"[Title/Abstract]) AND ("isoantibodies"[MeSH Terms] OR "alloantibod*"[Title/Abstract] OR "irregular antibod*"[Title/Abstract] OR "unexpected antibod*"[Title/Abstract] OR "red cell antibod*"[Title/Abstract] OR "erythrocyte antibod*"[Title/Abstract] OR "rbc antibod*"[Title/Abstract] OR "alloimmuni*"[Title/Abstract])	25 August 2025	984
Scopus	((TITLE-ABS-KEY ("Blood donor*")) OR (TITLE-ABS-KEY ("blood donation")) OR (TITLE-ABS-KEY ("voluntary donor*")) OR (TITLE-ABS-KEY ("first-time donor*")) OR (TITLE-ABS-KEY ("repeat donor*")) OR (TITLE-ABS-KEY ("donor screening"))) AND ((TITLE-ABS-KEY (alloantibod*)) OR (TITLE-ABS-KEY (isoantibod*)) OR (TITLE-ABS-KEY ("irregular antibod*")) OR (TITLE-ABS-KEY ("unexpected antibod*")) OR (TITLE-ABS-KEY ("red cell antibod*")) OR (TITLE-ABS-KEY ("erythrocyte antibod*")) OR (TITLE-ABS-KEY ("rbc antibod*")) OR (TITLE-ABS-KEY ("clinically significant antibod*")))	25 August 2025	1,095
Embase	[(blood donor/ or "blood donor*".mp.) OR ("blood donation".mp.) OR ("voluntary donor*".mp.) OR ("replacement donor*".mp.) OR (first time donor/ or "first-time donor*".mp.) OR (repeat donor/ or "repeat donor*".mp.) OR ("donor screening".mp.)] AND [(alloimmunization/ or alloantibody/ or alloantibod*.mp.) OR (isoantibody.mp.) OR ("irregular antibod*".mp.) OR (antibody screening/ or "unexpected antibody".mp. or erythrocyte antibody/) OR ("clinically significant antibody".mp.) OR ("RBC antibod*".mp.)]	25 August 2025	2,133
MEDLINE	[(blood donor.mp. or Blood Donors/) OR ("blood donation".mp. or Blood Donation/) OR ("voluntary donor*".mp.) OR ("replacement donor*".mp.) OR ("first-time donor*".mp.) OR ("repeat donor*".mp.) OR (" donor screening" . mp. or Donor Selection/)] AND [(alloantibody.mp. or Isoantibodies/) OR ("irregular antibody".mp.) OR ("unexpected antibody".mp.) OR ("erythrocyte antibody".mp.) OR ("RBC antibody".mp.) OR ("clinically significant antibody".mp.)]	25 August 2025	512
Google Scholar	RBC alloantibodies in blood donors	25 August 2025	113
