# Supplementary material for: Global Prevalence and Specificity of Red Blood Cell Antibodies in Blood Donors: A Systematic Review and Meta-Analysis
Source: Diseases. 2026 Aug 18;14(8):298. doi: 10.3390/diseases14080298 (PMC13512737; doi:10.3390/diseases14080298)
Supplement: Supplementary file 1 [file diseases-14-00298-s001.zip › Table S3. Characteristics of reports that remained unavailable in full text after retrieval attempts.pdf]

Supplementary Table S3. Characteristics of reports that remained unavailable in full text after retrieval attempts.

First author	Year	Title	Country	Language	Journal	Database identified	Retrieval attempts	Reason not retrieved
Aburto, A.	2021	Erythrocyte antigens and antibodies in the Chilean population.	Chile	English	Immunohematology	PubMed	Title-, author-, DOI-, PubMed, and publisher searches.	Publisher paywall; no accessible alternative full text found.
Bejrachandra, S.	1979	Unexpected red cell antibodies in donors and patients at Siriraj Hospital.	Thailand	English	Southeast Asian J. Trop. Med. Public Health	PubMed	Title-, author-, PMID-, PubMed, and journal/archive searches.	Abstract only; full text not available online and no accessible copy found.
Calkovská, Z.	1990	Frequency of Mg, Ny^a, Sc2, and Wr^a antigens and antibodies in central Slovakia.	Slovakia	English	Biomed. Biochim. Acta	PubMed	Title-, author-, PMID-, PubMed, and journal/archive searches.	Abstract only; full text not available online and no accessible copy found.
Huang, L.	2022	Results analysis of unexpected antibody screening for blood donors and cost comparison of two detection modes.	China	Chinese	Chinese Journal of Blood Transfusion	Scopus	Title-, author-, DOI-, Scopus, DOAJ, publisher, and CNKI searches.	Abstract available; full text inaccessible through the journal/CNKI platforms and no accessible alternative found.
Kout, M.	1980	Screening of allo-antibodies of blood groups in blood donors.	Czechoslovakia	Czech	Časopis lékařů českých	PubMed	Title-, author-, PMID-, PubMed, journal, and document-delivery searches.	Bibliographic record only; full text available only through paid document delivery and no accessible alternative found.
Li, Q.	2021	Prevalence of anti-Mi^a in blood donors and patients in Zhongshan City.	China	Chinese	Chinese Journal of Blood Transfusion	Scopus	Title-, author-, DOI-, Scopus, and CNKI searches.	Full text required institutional/IP access; no accessible alternative found.
Luo, Z.	2021	Profile of irregular antibodies among voluntary blood donors: 2018 to 2020 in Jiaxing.	China	Chinese	Chinese Journal of Blood Transfusion	Scopus	Title-, author-, DOI-, Scopus, and CNKI searches.	CNKI abstract available; full text required institutional/IP access and no accessible alternative found.

First author	Year	Title	Country	Language	Journal	Database identified	Retrieval attempts	Reason not retrieved
Matteocci, A.	1998	Screening and identification of clinically relevant irregular red blood cell antibodies: Comparison of two methods.	Italy	Italian	Trasfusione del Sangue	Scopus	Title-, author-, Scopus, DOI, journal, and database searches.	Scopus record/abstract only; journal full text not available online and no accessible alternative found.
Michael-Araszkiewicz, P.	1980	Automatic Marsh method for detection and identification of erythrocyte antibodies in patients and blood donors.	Poland	English	Archivum Immunologiae et Therapiae Experimentalis	PubMed	Title-, author-, PMID-, PubMed, and journal/archive searches.	Bibliographic record/abstract only; full text not available online and no accessible copy found.
Romphruk, A.V.	1999	Anti-P1: The most common unexpected antibodies in northeastern Thais.	Thailand	English	Journal of the Medical Association of Thailand	PubMed	Title-, author-, PMID-, PubMed, and journal/archive searches.	Detailed abstract available; full article not accessible and no alternative full text found.
