# Supplementary material for: Global Prevalence and Specificity of Red Blood Cell Antibodies in Blood Donors: A Systematic Review and Meta-Analysis
Source: Diseases. 2026 Aug 18;14(8):298. doi: 10.3390/diseases14080298 (PMC13512737; doi:10.3390/diseases14080298)
Supplement: Supplementary file 1 [file diseases-14-00298-s001.zip › Table S4. Characteristics and outcomes of included studies.pdf]

Table S4. Characteristics and Antibody Detection Outcomes of included studies

Study ID	Study	Country	Antibody detection method	Blood donors, n	Age, years	Sex (Male:Female)	Unexpected antibody-positive donors, *n*	Alloantibody-positive donors, *n*	Autoantibody-positive donors, *n*
1	Adewoyin, A.S. (2021)	Nigeria	CTT	80	29.04 ± 7.59	59 M:21 F	0	0	0
2	Akpan, I. (2022)	Nigeria	CTT	80	28 ± 7	36 M:44 F	0	0	0
3	Ameen, R. (2005)	Kuwait	CTT	170,197	N/A	N/A	30	30	0
4	Arabi, S. (2021)	Iran	CTT	358	18–68	329 M:29 F	0	0	0
5	Babaei, K. (2016)	Iran	CTT	75	19-66	73 M:2 F	6	6	0
6	Bharathan, P. (2019)	India	CAT	2,310	30.3	2,299 M:11 F	1	1	0
7	Chen, L. (2020)	China	CTT and CAT	72,684	18–55	49,132 M:23,552 F	55	48	7
8	Dong, P. (2023)	China	Microplate and CAT	12,293	N/A	N/A	6	6	0
9	Ducos, J. (1978)	France (Paris)	Automated Groupamatic	661,511	N/A	N/A	2,118	2,118	0
10	Ducos, J. (1978)	France (Toulouse)	Automated Groupamatic	269,162	N/A	N/A	4,302	2,145	2,157
11	Dueñas, R.V.H. (1999)	Colombia	CTT	45,127	18–65	27,302 M:17,825 F	177	177	0
12	Hashem, A.E.A. (2018)	Egypt	CTT	200	32	130 M:70 F	8	8	0
13	Erikstein, B.S. (2019)	Norway	CAT	2,441	31	N/A	3	3	0
14	Garg, N. (2014)	India	CAT	47,450	18–55	47,065 M:385 F	56	50	6
15	Garozzo, G. (2007)	Italy	SPRCA	560	N/A	N/A	4	4	0
16	Gupta, C. (2019)	India	CAT	1,999	N/A	N/A	13	13	0
17	Karafin, M.S. (2019)	USA	SPRCA, CTT or CAT	632,378	16–80	314,806 M:317,566 F	4,861	4,861	0

Study ID	Study	Country	Antibody detection method	Blood donors, n	Age, years	Sex (Male:Female)	Unexpected antibody-positive donors, *n*	Alloantibody-positive donors, *n*	Autoantibody-positive donors, *n*
18	Kaur, D. (2017)	India	CAT	6,350	N/A	6,172 M:178 F	7	4	3
19	Keokhamphoui, C. (2014)	Laos	CTT	1,181	N/A	821 M:360 F	46	46	0
20	Luhar, R.K. (2022)	India	SPRCA and EM technology	53,724	N/A	51,185 M:2,539 F	25	25	0
21	Makroo, R.N. (2018)	India	SPRCA	82,153	18->40	81,193 M:960 F	227	77	150
22	Malhotra, S. (2020)	India	CTT and CAT	10,390	32.31± 10.05	9,959 M:431 F	19	19	0
23	Myhre, B.A. (1965)	USA	CTT	187,901	N/A	N/A	2,498	640	0
24	Nathani, K. (2021)	India	CTT and CAT	29,105	18-60	28,613 M:492 F	7	6	1
25	Niyibizi, J.B. (2025)	Rwanda	EM Technology	8,693	N/A	N/A	10	10	0
26	Pahuja, S. (2012)	India	CAT	7,756	18->40	7,648 M:108 F	4	3	1
27	Patel, J. (2022)	India	EM Technology	216,576	18->40	200,493 M:16,083 F	66	58	8
28	Plumbley, J.A. (1999)	USA	Microplate and CTT	38,436	18–22	N/A	14	14	0
29	Pons, S. (2023)	France	SPRCA	212,218	32 ± 12	157,898 M:54,320 F	131	111	20
30	Promwong, C. (2013)	Thailand	CTT	20,786	N/A	N/A	183	183	0
31	Promwong, C. (2013)	Thailand	CAT	49,229	N/A	N/A	98	98	0
32	Randjelović, M. (2024)	Serbia	Microplate and CAT	23,082	48.81 ± 9.46 (Positive donors)	19,358 M:3,724 F	26	26	0
33	Rastogi, V. (2025)	India	CAT	2,500	18–50	2,225 M:275 F	4	4	0
34	Sachan, D. (2019)	India	CAT	40,629	N/A	38,878 M:1,751 F	57	41	16

Study ID	Study	Country	Antibody detection method	Blood donors, n	Age, years	Sex (Male:Female)	Unexpected antibody-positive donors, *n*	Alloantibody-positive donors, *n*	Autoantibody-positive donors, *n*
35	Sallander, S. (1996)	Sweden	SPRCA	3,778	N/A	N/A	17	17	0
36	Santos, L.D.S. (2024)	Brazil	SPRCA	205,965	33.2± 10.8	121,556 M:84,409 F	753	753	0
37	Shafini, M.Y. (2017)	Malaysia	CAT	500	N/A	422 M:78 F	4	4	0
38	Solanki, A. (2020)	India	EM Technology	166,803	18–65	156,128 M:10,675 F	286	256	30
39	Tiwari, A.K. (2014)	India (Northern India)	CAT	31,367	18–60	29,979 M:1,388 F	16	3	13
40	Toledo, M.E.R. (2019)	Colombia	NA	35,248	N/A	N/A	71	67	4
41	Yadav, A. (2022)	India (Northern India)	CAT	2,000	N/A	1,643 M:357 F	6	4	2
42	Yang, H. (2024)	China (Changzhou)	CTT and CAT	190,780	N/A	111,589 M:79,191 F	186	186	0
43	Zhu, J.Y. (2007)	China (Shaoguan)	NA	15,033	18–55	9,220 M:5,813 F	42	42	0

Abbreviations: CAT, column agglutination technique; CTT, conventional tube testing; EM, erythrocyte magnetized technology; NA, not available; SPRCA, solid-phase red cell adherence assay.

Notes: Unexpected antibody-positive donors include donors with alloantibodies, autoantibodies, or both, as reported in the original studies. Antibody screening methods are presented as described by the original authors. Two studies (Ducos et al. and Promwong et al.) reported independent donor subgroups and were treated as separate reports in the meta-analysis.
