# Supplementary material for: Global Prevalence and Specificity of Red Blood Cell Antibodies in Blood Donors: A Systematic Review and Meta-Analysis
Source: Diseases. 2026 Aug 18;14(8):298. doi: 10.3390/diseases14080298 (PMC13512737; doi:10.3390/diseases14080298)
Supplement: Supplementary file 1 [file diseases-14-00298-s001.zip › Table S5. Quality assessment of included studies .pdf]

Table S5. Quality assessment of included studies

No.	Study	Q1	Q2	Q3	Q4	Q5	Q6	Q7	Q8	Q9	Overall quality
1	Adewoyin, A. S. (2021)	Green	Green	Yellow	Green	Green	Green	Green	Green	Green	High
2	Akpan, I. (2022)	Green	Green	Yellow	Green	Green	Green	Green	Green	Green	High
3	Ameen, R. (2005)	Green	Green	Green	Red	Green	Green	Green	Green	Green	High
4	Arabi, S. (2021)	Yellow	Yellow	Yellow	Green	Green	Green	Green	Green	Green	Moderate
5	Babaei, K. (2016)	Green	Green	Yellow	Yellow	Green	Green	Green	Yellow	Green	Moderate
6	Bharathan, P. (2019)	Green	Green	Green	Red	Green	Green	Green	Red	Green	High
7	Chen, L. (2020)	Green	Green	Green	Red	Green	Red	Green	Green	Green	High
8	Dong, P. (2023)	Yellow	Yellow	Green	Yellow	Green	Green	Green	Yellow	Green	Moderate
9	Ducos, J. (1978)	Green	Green	Green	Green	Green	Red	Green	Yellow	Red	High
10	Dueñas, R.V.H. (1999)	Green	Green	Green	Green	Green	Green	Green	Yellow	Green	High
11	Hashem, A.E.A. (2018)	Green	Green	Yellow	Green	Green	Green	Green	Yellow	Green	High
12	Erikstein, B.S. (2019)	Green	Green	Green	Green	Green	Green	Green	Yellow	Green	High
13	Garg, N. (2014)	Green	Green	Green	Green	Green	Green	Green	Yellow	Green	High
14	Garozzo, G. (2007)	Green	Green	Yellow	Green	Yellow	Green	Green	Yellow	Green	Moderate
15	Gupta, C. (2019)	Green	Green	Yellow	Green	Yellow	Green	Green	Yellow	Green	Moderate
16	Karafin, M.S. (2019)	Green	Green	Green	Red	Yellow	Green	Green	Green	Green	High
17	Kaur, D. (2017)	Green	Green	Green	Red	Green	Green	Green	Yellow	Green	High
18	Keokhamphoui, C. (2014)	Green	Green	Yellow	Green	Green	Green	Green	Green	Green	High
19	Luhar, R.K. (2022)	Green	Green	Green	Red	Yellow	Green	Green	Yellow	Green	Moderate
20	Makroo, R.N. (2018)	Green	Green	Green	Green	Green	Green	Green	Red	Green	High
21	Malhotra, S. (2020)	Green	Green	Green	Red	Green	Green	Green	Green	Green	High
22	Myhre, B.A. (1965)	Green	Green	Green	Green	Yellow	Yellow	Green	Yellow	Red	Moderate
23	Nathani, K. (2021)	Green	Green	Green	Red	Green	Green	Green	Yellow	Green	High
24	Niyibizi, J.B. (2025)	Green	Green	Green	Green	Green	Green	Green	Green	Green	High
25	Pahuja, S. (2012)	Green	Green	Green	Green	Green	Green	Green	Red	Green	High
26	Patel, J. (2022)	Green	Green	Green	Green	Green	Green	Green	Yellow	Green	High
27	Plumbley, J.A. (1999)	Green	Green	Green	Green	Green	Yellow	Yellow	Yellow	Green	Moderate
28	Pons, S. (2023)	Green	Green	Green	Green	Green	Green	Green	Red	Green	High
29	Promwong, C. (2013)	Green	Green	Green	Green	Green	Green	Green	Yellow	Green	High
30	Randjelović, M. (2024)	Green	Green	Green	Red	Green	Green	Green	Yellow	Green	High
31	Rastogi, V. (2025)	Green	Green	Green	Red	Green	Green	Green	Yellow	Green	High
32	Sachan, D. (2019)	Green	Green	Green	Red	Green	Green	Green	Yellow	Green	High
33	Sallander, S. (1996)	Green	Green	Green	Red	Green	Green	Green	Yellow	Green	High
34	Santos, L.D.S. (2024)	Green	Green	Green	Green	Yellow	Green	Green	Green	Green	High
35	Shafini, M.Y. (2017)	Green	Green	Yellow	Green	Green	Red	Green	Yellow	Green	Moderate
36	Solanki, A. (2020)	Green	Green	Green	Green	Green	Green	Green	Green	Green	High
37	Tiwari, A.K. (2014)	Green	Green	Green	Red	Green	Green	Green	Green	Green	High
38	Toledo, M.E.R. (2019)	Green	Green	Green	Red	Green	Red	Green	Green	Green	High
39	Yadav, A. (2022)	Green	Green	Green	Red	Green	Green	Green	Green	Green	High
40	Yang, H. (2024)	Green	Green	Green	Green	Green	Green	Green	Green	Green	High
41	Zhu, J.Y. (2007)	Green	Green	Green	Yellow	Green	Red	Green	Yellow	Green	Moderate

Each study was assessed across nine methodological domains (Q1–Q9). Responses were recorded as Yes, No, or Unclear, and overall study quality was categorized as high, moderate, or low based on the number of domains rated as "Yes".
